# Neurocognitive differences in sketching between design tasks and creativity tests

**DOI:** 10.1038/s41598-026-38735-w

**Published:** 2026-02-20

**Authors:** Shumin Li, Gaetano Cascini, Niccolò Becattini

**Affiliations:** Department of Mechanical Engineering, Politecnico di Milano, 20158 Milan, Italy

**Keywords:** Human behaviour, Neurophysiology, Engineering, Cognitive neuroscience

## Abstract

This study investigates differences in brain activity among thirty-seven engineering students during sketching in a creativity test and a design task. Using EEG data, we first examined the impact of baseline selection (eyes-open vs. eyes-closed) on the representation of results in terms of de/synchronization across frequency bands from theta (4–7 Hz) to lower gamma (30-45 Hz). Specifically, an eyes-closed baseline allowed for the observation of alpha desynchronization during sketching, whereas an eyes-open baseline, which better aligns with real-world design conditions, shifted the pattern to synchronization. Under the eyes-open baseline, the comparisons between the two sketching tasks revealed the following results: (1) regions that showed significant bilateral TRP asymmetries in creativity test sketching also exhibited differences in design sketching across frequency bands from theta to beta band. Areas that exhibited significant bilateral TRP asymmetry only in design sketching were located in the temporal sites (theta and beta), suggesting these areas within the corresponding frequency bands could serve as indicators for distinguishing between the two cognitive tasks. (2) Statistically significant differences between the two sketching tasks in channel comparisons were primarily observed in the theta and sub-alpha bands, especially in the left frontal and right hemisphere areas. (3) The beta band exhibited similar behavior across both sketching tasks, indicating shared cognitive processes in different sketching contexts. (4) While the lower gamma band did not show significant differences in channel comparisons between tasks, it exhibited distinct bilateral TRP asymmetry in the frontocentral area only in the creativity test, highlighting the potential of bilateral asymmetry as a more sensitive measure for distinguishing between design and creativity tasks.

**Supplementary Information:**

The online version contains supplementary material available at 10.1038/s41598-026-38735-w.

## Introduction

In the design domain, sketching has long been acknowledged as a fundamental and powerful tool in the ideation and conceptualization processes, as it externalizes mental representations into visual forms and supports idea exploration and refinement^1–3^.

Much of the design literature has examined how creativity is manifested during sketching^4,5^, by means of sketching^6,7^, and through the outcomes of sketching^8,9^, often through observational approaches that inherently introduce subjectivity and pose interpreting challenges in unfolding the underlying cognitive behavior during sketching. To overcome these limitations, neurophysiological methods (e.g., EEG) have increasingly been adopted to study creativity and design cognition^10,11^. However, these investigations are mainly based on standard creativity tests rather than actual design tasks^12^. In particular, the Torrance Test of Creative Thinking-Incomplete Figures (TTCT-IF^13^) has been widely employed to study visual creativity^14,15^. While informative, TTCT-IF emphasizes figural imagination and sketching activities under simplified and controlled conditions. This raises questions about whether the neural patterns observed in TTCT-IF can be generalized to design sketching, which is often constrained by functional requirements, requires integrative composition of multiple features, and demands more complex reasoning and visuospatial integration^16,17^. The present study aims to address this gap by investigating whether cognitive activities during different sketching tasks can be distinguished through electroencephalogram (EEG) data, specifically comparing sketching in a design task and sketching in a standard creativity test (i.e., TTCT-IF).

Following the introduction section, this paper first presents the background of the study. Then, it describes the employed methodology, detailing the experimental protocol, signal processing algorithms, and how the statistical hypothesis testing provides elements to answer the research question. The results section then showcases the EEG behavior observed from the experiments. The paper then discusses these results and concludes with a final section summarizing the study’s key findings.

## State of the art

Sketching and sketches are crucial in various stages of the design process^18,19^. By promoting information processing and externalizing the designer’s ideas, sketching can serve as a cognitive tool to support design reasoning^20^. It facilitates rapid processing of spatial and visual information and enhances memory and thought^21^. It can store the created idea while simultaneously offering the designers immediate feedback^22^, providing the designer with new insights that stimulate further ideas^23^, and boosting exploration’s fluidity^19^. Sketches may enhance fixation effects^24^ by reducing the variety and originality of ideas generated during creativity sessions^25^. Research on design cognition demonstrated that novice sketchers are not more creative when they sketch and that the effectiveness of sketches to increase creativity greatly depends on the design strategy followed by the designer^5^.

Sketches are used in both divergent thinking and convergent thinking, which are commonly recognized design reasoning processes involved in idea exploration^19^. Divergent thinking fosters diversity and exploration in idea generation, resulting in a wider variety of screened possibilities, while convergent thinking helps focus and refine ideas. During sketching, there might be a dynamic interplay of convergent and divergent thinking, which enables designers to navigate the complexity of the design space effectively. A good design often results from effectively balancing both processes. However, novice designers may find it challenging to apply this balance in their early practice^21^. Recent studies focus on integrating neurophysiological data with traditional observational approaches to distinguish different cognitive processes across various types of creativity tests^14,26,27^. This could potentially allow the design process to be monitored and guided in real-time using biofeedback, which may reduce the need for additional guidance during the ideation process for novice designers. However, unlike creativity studies, there has been limited exploration of the design activities incorporating sketching through neuro-physiological measures. Furthermore, it has been discovered that sketching differs cognitively from other design tasks (e.g. verbalization^28^ and typing^29^); hence, the findings from other studies cannot be applied directly to sketching-based design activities.

Recent reviews^10,11^ indicate that the neurophysiological signal measurements mainly employed in design cognition studies include: functional magnetic resonance imaging (fMRI), which necessitates that individuals remain motionless in the scanning tunnel until data collection is complete; near-infrared spectroscopy (NIRS), which is restricted to the frontal areas of the scalp, with interpretation challenges related to multiple sources of the vascular signal; and electroencephalogram (EEG), which primarily reflects scalp-level brain activity, while sensitive to various sources of noise, it offers excellent time resolution and greater flexibility for body movements compared to fMRI. Additionally, EEG typically covers more areas of the scalp than NIRS, providing a more comprehensive dataset for analysis. Of all the available techniques, EEG measurement was considered the most appropriate for the current investigation given its strengths and the presence of existing research evidence to evaluate the data quality.

Activities involving sketching as part of creativity tests (mostly in psychology studies) or freehand design sketching have previously drawn attention and have been investigated by several EEG data-based research studies. The review by Pidgeon et al.^11^ provided insights into visual creativity, specifically how EEG was used to examine brain activity during creativity tests involving sketching. Visual creativity, in this context, refers to generating new and practical visual ideas by transforming mental images into visual forms through sketching or painting. The review found that the task most commonly employed in EEG-based studies was the Torrance Test of Creative Thinking-Incomplete Figures (TTCT-IF^13^). The task requires the participant to generate a complete image from a provided fragment of a drawing (i.e., basic geometric shapes like lines, circles, and rectangles). Most of the reviewed contributions considered EEG power (POW), or event/task-related power (ERP/TRP) and EEG coherence (COH) of lower alpha (alpha1: 7–10 Hz) and upper alpha (alpha2: 10–13 Hz) sub-frequency bands. Although the observations were not always consistent within each measure (power or coherence), the studies utilizing power analysis revealed a more pronounced trend in brain signal behavior compared to those focusing on coherence. Table 1 presents the studies selected from the review, along with additional studies that focus on EEG-based analysis of the TTCT-IF test, which considered the use of TRP or POW as (part of) the data treatment method.

**Table 1:** Research findings based on the TTCT-IF test.

Author	Cohort (final sample that performed TTCT)	Baseline Activity	TTCT duration	Frequency Band	Treatment (POW/TRP/COH)	Result (Snyc/Desnyc)
W. Jia & Y. Zeng (2021)^14^	28 Information Systems Engineering graduate students (age 22-35, 4 females)	3-min eyes-closed	3-min TTCT sketching	$\alpha 1$, $\alpha 2$	mean TRP	$\alpha 1$: desynchronization across all the areas, stronger at the parietal sites compared to frontal; $\alpha 2$: desynchronization across all the areas, lower at temporal sites compared to occipital sites.
C. Rominger et al. (2018)^15^	50 participants (48 university students, age 19-42, 32 females); unspecified background	15-sec eyes-open	8-sec mental idea generation + 8-sec idea mental elaboration	$\alpha 2$	median TRP	$\alpha 2$: Strong desynchronization over parietal and occipital sites.
N. V. Volf & I. V. Tarasova (2014)^30^	31 students from higher educational institutions in Novosibirsk (aged 18–21 years, 16 females); unspecified background	2-min eyes-open	3-min mental idea generation with vs without monetary rewards	$\theta 1$, $\theta 2$, $\alpha 1$, $\alpha 2$, $\beta 1$, $\beta 2$	POW	$\theta 1$: desynchronization, higher TRP in the reward condition; $\alpha 1$ and $\alpha 2$: desynchronization, higher TRP in the non-reward condition; $\beta 1$ and $\beta 2$: desynchronization, especially in the posterior brain areas.
N. V. Volf & I. V. Tarasova (2010)^31^	28 students from higher educational institutions in Novosibirsk (aged 18–21 years, 14 females); unspecified background	1-min eyes-open	1-min mental idea generation, then verbalize the ideas, sketch at the end	$\theta 1$, $\theta 2$,$\beta 1$,$\beta 2$	POW & TRP	$\theta 2$: desynchronization in the frontal-occipital area (high-originality men, not low-originality men or women); $\beta 1$: desynchronization in men, synchronization in women. High creative efficiency women have higher $\beta 1$ power in caudal regions and opposite asymmetry patterns to high creativity men.
O. M. Razumnikova et al. (2009)^32^	26 students from higher educational institutions in Novosibirsk (total sample: 53 students, aged 18–21 years, 26 females); unspecified background	30-sec eyes-open	1-min mental idea generation	$\theta 1$, $\theta 2$, $\alpha 1$, $\alpha 2$, $\beta 1$, $\beta 2$	TRP & COH	$\theta 1$: desynchronization in the figural task (synchronization in the verbal task); $\alpha 1$:desynchronization; $\alpha 2$: desynchronization (more reactive in women in verbal tasks); $\beta 2$: synchronization, more reactive in men in figural tasks. Right hemisphere activity dominated during creative tasks across sexes.
N. Jaušovec & K. Jaušovec (2000)^28^	30 student-teachers taking a course in Psychology (age 18-19, 18 females)	5-min eyes-open	2-min mental idea generation	$\alpha 1$, $\alpha 2$	POW & COH	$\alpha 1$ and $\alpha 2$: desynchronization.

All the studies presented in Table 1, except the recent work by Jia and Zeng^14^, instructed participants to generate sketching ideas mentally, focusing their EEG analysis on the imaginative phase. As a result, these studies primarily emphasized figural imagination abilities rather than the actual progression of idea development through sketching. A common observation in this body of research was task-related alpha desynchronization when compared to the baseline period. This desynchronization was also observed in Jia and Zeng’s study^14^. However, it is important to note that Jia and Zeng used an eyes-closed period as the baseline, which differs significantly in power from the eyes-open condition^33^. Notably, the eyes-open condition could offer greater continuity and comparability with task activities like sketching, as it more closely reflects the neural state associated with visual engagement and active cognitive processing during such task activities. This highlights a gap in the literature, as studies have yet to use an eyes-open baseline while incorporating actual sketching as the test activity. Therefore, the first research question this paper would like to address is what the de/synchronization brain behavior of engineering students when sketching in TTCT-IF with reference to the different eye conditions used as a baseline for TRP estimation is, and how these observations span a wider frequency range.

Most of these studies have primarily focused on the alpha band due to its well-established correlation with creativity, while notable results were also observed in the beta band^30–32^. However, higher frequencies, such as the gamma band, have not been reported in any studies using the TTCT-IF task. On the one hand, the existing findings about the alpha band in sketching activities require extending the analysis to EEG behavior using the eyes-open baseline condition. On the other hand, broadening the analysis to include more frequency bands can offer a more comprehensive understanding of brain behavior in the accomplishment of this task and more comparison opportunities with data collected in other relevant tasks. Additionally, the participants in these studies were from different backgrounds (mostly in psychology), raising the question of whether the observations of sketching activities in the TTCT-IF test can be generalized to design sketching that requires specialized technical knowledge (e.g. engineering).

Regarding the EEG-based research that focuses on design sketching rather than creativity tests, through a series of studies, Vieira et al. compared freehand design sketching within an open design task with a constrained problem-solving design task from diverse viewpoints^17,34^. Their research demonstrated that EEG data exhibit a main effect of gender in the theta, higher alpha, and lower beta frequency bands. In these frequency bands, females show higher activation in the right dorsolateral prefrontal cortex, right occipitotemporal cortex, secondary visual cortex (i.e., part of the occipital cortex), and prefrontal cortex on both tasks. Both genders show higher power in the theta and alpha1 band in the right hemisphere and higher power in the alpha2 band and the beta band across hemispheres during the open design sketching task^34^. Subsequently, they found significant differences between constrained and open designs for the EEG power in beta bands at the earliest reaction stage immediately after reading the request. Then, the alpha2 band and beta band were found to have significant differences between the two tasks in the stage of sketching to externalize the solution^17^. Despite numerous studies on sketching-based creativity tests and some efforts in sketching for design, there remains a significant gap in research focusing on neurocognitive behavior during sketching and visual creativity within design contexts. The current literature lacks sufficient exploration of how brain activity during sketching varies across different scenarios also raises the question of whether findings from creativity tests can be reliably used to interpret design cognitive behavior through EEG measurements.

This difficulty in generalizing findings becomes even more pronounced when considering the differences in context, objectives, and cognitive demands between structured creativity tests and real-world design tasks. The contrast between sketching in creativity tests and design is consistent with dual-process in cognitive psychology^35,36^, which distinguishes human cognition as being governed by two systems (i.e., system 1: fast, intuitive and effortless thinking; system 2: slower, analytic thinking). Sketching in a creativity test such as TTCT-IF is open-ended and primarily divergent. It emphasizes intuitive associative processing (system 1), imposes no functional requirements on the drawing and does not set a specific outcome goal for the sketch^37^. The constraints are task-based, arising only from the fragments provided for elaboration. In contrast, design sketching is goal-oriented and context-dependent. To propose a novel design concept by means of sketching, the designer should integrate multiple partial solutions into a coherent whole that satisfies explicit functional requirements under practical constraints. This process involves analytic processing (system 2), iterative evaluation of trade-offs, and a strong balance between divergent and convergent thinking^38^. Compared with creativity tests, these additional constraints increase cognitive load by introducing higher demands on problem-solving, visuospatial reasoning and decision making^17,21^. Furthermore, it also aligns with design frameworks that describe design as complex, iterative activities of idea generation and evaluation rather than a pure intuitive thinking process^3,39^. Therefore, it is crucial to investigate sketching activities in both contexts to better understand the cognitive processes involved and to determine the applicability of creativity test findings to real-world design tasks. By analyzing the neurophysiological data, such as EEG in this study, we intend to explore cognitive differences in design activities that utilize the same idea expression modalities as those used in creativity tests (e.g., sketching). This exploration may also shed light on the ongoing debate about the differences between design creativity and creativity measured by divergent thinking tests.

In light of the previously identified research gaps, the present study aims to address the use of inconsistent baselines, non-comprehensive frequency band analysis, participant background diversity, and challenges in generalizing results. Specifically, it focuses on distinguishing and comparing EEG behaviors observed during sketching tasks performed by the same group of engineering students. By employing an eyes-open condition as the baseline and analyzing EEG data across a broader range of frequency bands, this study aims to provide a more comprehensive understanding of cognitive processes in sketching for different purposes. Through this approach, the study seeks to answer the following research questions:

- RQ1: What is the de/synchronization brain behavior of engineering students when sketching in TTCT-IF with reference to the different eye conditions used as a baseline for TRP estimation?
- RQ2: How does the brain behavior of engineering students differ when sketching in a design task and in the TTCT-IF test?

The methodology used to address these research questions is presented in the following section.

## Methods

To address the research questions, we designed an experiment involving two sketching-based tasks: the TTCT-IF task and a design task in which participants were required to sketch a unique design concept based on specific design requirements. These tasks were part of a larger experiment that also included other design- or creativity-related tasks. To minimize the effects of mental fatigue, the tasks were presented to the participants in random order. The current study uses an existing dataset, as for^40^, which is composed by EEG data collected during the execution of different tasks. The data overlapping this and previous studies is limited to the EEG data collected during the sketching activity in the design task - previous studies compared that task with other design-related activities^40^. The current study, however, addresses different research questions and therefore relies on additional data which were not previously considered for analyses and comparisons.

At the beginning of the experiment, participants underwent a gaze fixation session (referred to as eyes-open throughout the rest of the paper) and an eyes-closed relaxation session. The EEG recordings taken from these sessions facilitated the statistical comparison of TRP values in TTCT-IF using different baseline activities to address the first research question via hypothesis testing. Then, using the eyes-open session as the baseline for calculating TRP values, we examined and compared brain activity during the two sketching tasks to address the second research question. This was done in two further hypotheses checks: (a) checking for statistically significant differences in bilateral asymmetry between each pair of channels, and comparing whether the results differed between the two sketching tasks; (b) conducting a channel-to-channel comparison between the two tasks to determine if any statistically significant differences were present.

### Material

The experimental process was programmed via Psychopy 3^41^, whose log file enabled the synchronization of the whole dataset (EEG, video recording, and keyboard operation). The participants received information about the task and its execution presented on the computer screen and could ask clarification questions before starting each task. In addition to the task instructions displayed on the computer, participants were given paper-format material to work with when the task involved sketching activities.

**Fig. 1:**
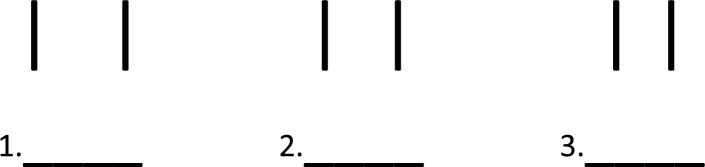
TTCT-IF test in the current study.

The TTCT-IF task asks participants to add elements to given simple geometric patterns, ensuring that the completed drawings are meaningful by assigning names to clarify their ideas. There are many sample versions of incomplete figures available. The set used in this study is shown in Fig. 1. Three rows of the same set of patterns were shown (9 incomplete figures in total) on the A4 paper provided to each participant. Additional papers with the same set of incomplete patterns could be provided in case the participants completed the sketches before the time was over.

The design task in this study asks the participants to develop a unique concept of an amphibious bike (a human-powered vehicle that can operate on both land and water) using a given morphological table, namely Design with Morphological Table (DwMT). To accomplish this task, the participants must first read the task instructions and choose one partial solution from the given options for each feature (Table 2), considering the following three design requirements: the amphibious bike should be able to (1) Support at least 2 people (> 160 kg); (2) transition time between water and land mode: less than 2 min; (3) be transportable on vehicle bike racks or in trunks (length < 4m). The complete table with accompanying images of the feature options is the same as the one used in previous studies^40^. Upon completing the selection of partial solutions, participants were instructed to merge them all into a single concept and draw it on paper.

**Table 2:** The text content of the morphological feature table for designing an amphibious bike.

Features	a	b	c	d
Number of wheels	0	1	2	3
Wheel type	Inflatable bicycle tyre	Airless tyre	Rigid floating tyre	
Propulsion in water	Propeller	Paddle wheel	Paddles	
Number of propulsion units	0	1	2	3
Powered by	Hand	Foot	Both	
Riding position	Recumbent seating	Upright seating	Upright standing	
Seat arrangement	Side by side	Fore to aft		
Steering	Flexible shaft	Propeller	Rudder	
Power transmission	Chain	Drive shaft	Belt	
Frame material	Aluminum	Composite(e.g. carbon fiber)	Plastic	
Buoyancy	Inflatable elements	Hull	Rigid float	

### Procedure

All the experiments were conducted in the same sound-attenuated laboratory during the same season. The illumination and the ambient temperature of the room were consistently maintained during the experiment sessions. Participants were instructed to maintain a sitting position during the whole trial and refrain from making frequent or continuous body movements. All the experiment sessions were audio/video recorded to assist further data-processing procedures.

Before the experiment began, the participants were asked to read the general introduction of the research project and provide consent to measure and record their data for research purposes. The experiment manager then verbally presented additional information, such as the task’s nature, how to interact with the program, the experiment duration, etc. At the same time, the experiment manager started assisting the participant put on the EEG headset. The participants were then invited to complete a short demographic survey and took some time to adjust to the headset.

Once the recording began and the participants agreed to proceed with the experiment, they were advised to keep their eyes open for 30 seconds while gazing at a fixation cross in the middle of the screen, followed by closing their eyes for an additional 30 seconds. The participants then proceeded through five tasks of the whole experiment. Each task was preceded by an adapted version of the Wisconsin Card Sorting Test (WCST^42^) that lasted for 36 seconds (at most). In WCST, the participant had to select the only card out of four that shared the same feature (i.e., shape, color, number of patterns) with a randomly displayed card. In the original WCST, the matching features change unpredictably without direct indication, and participants must figure out the correct feature through corrective feedback. In contrast, the test used in the current experiment pre-informed the participants of the feature that they should consider helping the participants detach from the demands of the previous task without creating an excessive mental workload.

After the completion of each round of the WCST game, the participants accessed the instructions for the next task, together with an on-screen example. Meanwhile, the participants might ask questions and receive more explanations regarding the task at hand. There was no time constraint for reading the instructions, and the tasks began only after participants indicated they were ready by pressing the space button. The duration of the TTCT-IF task was three minutes. The information on the remaining time was shown on the screen throughout the ongoing progress. The participants got two audio reminders, one to inform them that the task entered the last minute and another at the end of the allotted time. Similar audio notifications on the remaining time were also given for the ten-minute DwMT task, with an additional one when half-time had passed. The participants were provided with the corresponding sketching papers (size A4) after confirming their understanding of the task, and then the timer began. While the TTCT-IF task encourages participants to sketch continuously throughout the allotted time to create as many meaningful figures as possible, sketching in the DwMT occurs primarily in the final portion of the DwMT task, following the task’s structured sequence. The activities in DwMT were recognized based on the video recording: in the initial phase, the participants explored the morphological table and selected their favorite options; then, they started the sketching activity: the transition was recognized at the exact moment when they drew their first line on the paper and ended when the time ran out (or they switched to the next experiment section). The video recording provides evidence of the participants’ full commitment to sketching the design concept during the initial two minutes once they start drawing, after which they may review the choices and possibly make modifications. Hence, the present investigation just focuses on the initial two minutes of sketching in DwMT. The drawing step of the DwMT will be referred to as DwMTs in subsequent portions of this article.

### EEG recording and processing

**Fig. 2:**
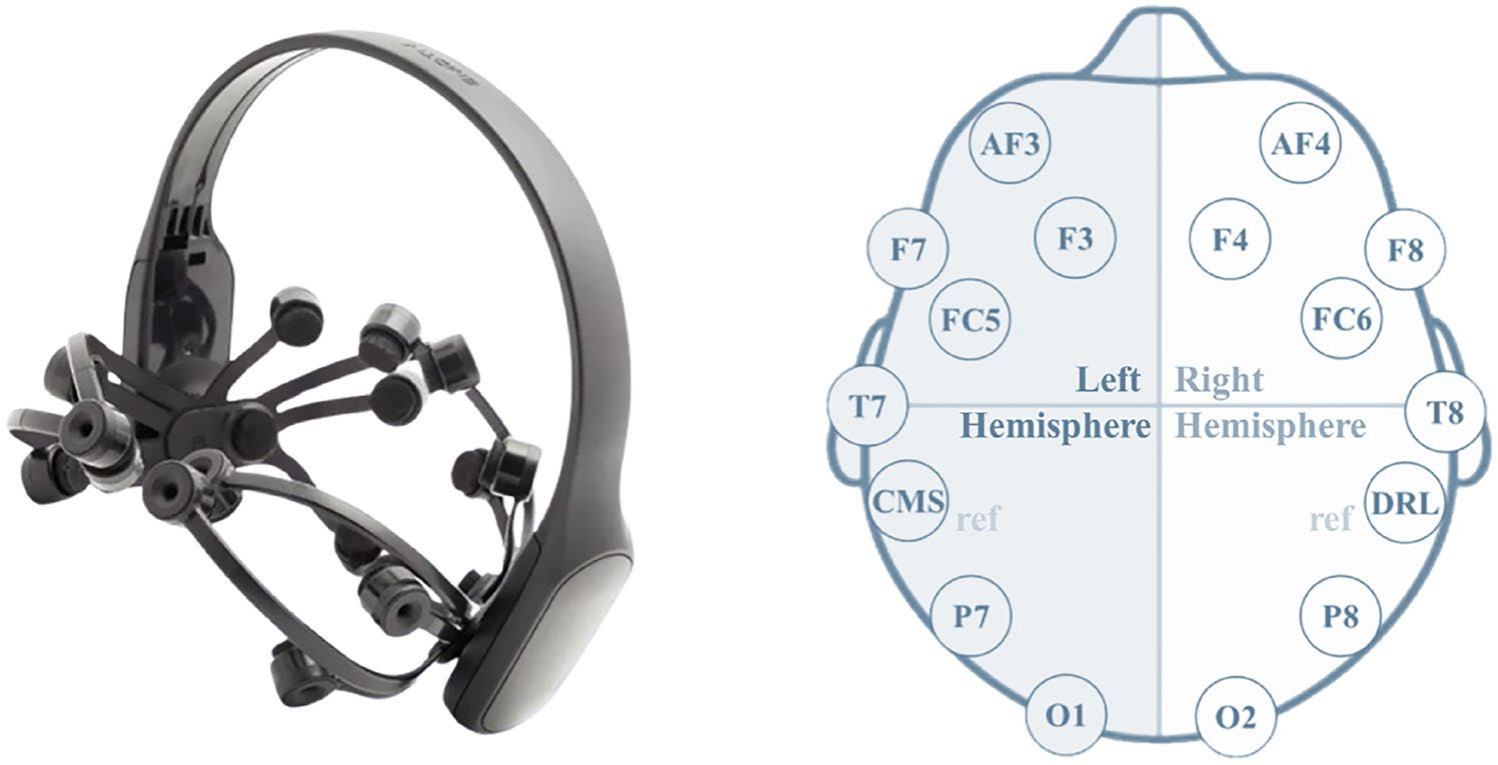
Emotiv EPOC X headset and the 14 sensors configuration.

This study was conducted using the Emotiv EPOC X headset. The headset features a pre-mounted frame with 14 electrodes (AF3/4, F7/8, F3/4, FC5/6, T7/8, P7/8, and O1/2) arranged according to the International 10-20 system, forming seven pairs of symmetric channels, as shown in Fig. 2. Two additional electrodes (CMS/DRL) serve as references. Although it offers lower spatial coverage than high-density systems, prior work shows that EMOTIV headsets can provide a good balance between usability and coverage for ecologically valid recording^43,44^. Similar configurations have already produced meaningful findings in design and creativity relevant contexts^17,45,46^. The sampling frequency was set at 128 Hz. This sampling frequency allowed the analysis of the data bands up to the lower gamma band ([30-45] Hz), according to the Nyquist-Shannon sampling theorem. However, the signal-noise ratio (i.e., noise that cannot be removed by signal processing methods) in the delta band ([0-4]Hz) was noticeably low in the collected data. As a result, the final analysis of the current study focused on the frequency bands between 4 and 45 Hz (i.e., theta [4–7] Hz, alpha1 [7–10] Hz, alpha2 [10–13] Hz, beta [13–30] Hz, and lower gamma [30–45]Hz bands). The raw data were extracted from EmotivPRO application for processing and analysis in accordance with the current research needs.

**Fig. 3:**
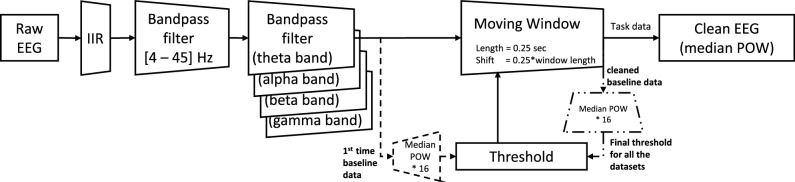
The signal-processing pipeline.^40^.

EEG signals are sensitive to many interfering sources. The electromyographic signal evoked by movements of the eye, neck, and face muscles, as well as the signal interruption brought on by a drop in contact quality, were typical artifacts in our study. To produce a clean dataset for the analysis, their removal was essential. Component analysis-based signal cleaning algorithms like independent component analysis^47^, principle component analysis^48^, and canonical correlation analysis^49^ are commonly used in clinical EEG-based studies but can introduce biases in TRP-based analyses, especially for datasets with unequal artifact numbers and distributions. These algorithms remove unbalanced signal power in the reference activity and the task activity, affecting the analysis of results. Therefore, the signal processing approach which has been already used in the literature^40^ was employed in the current study to process the EEG signal (Fig. 3). This approach involved applying a standard FFT band-pass filter to the raw data as the first step, followed by a logical distinction of artifacts referenced individually to reject the contaminated segments.

The pipeline initially uses an infinite impulse response (IIR) filter to eliminate the DC offset that the headset consistently added to the raw data during pre-processing. The band-pass filter then cuts off the frequencies that are not within the range of [4–45] Hz, as mentioned earlier. Subsequently, the data is subjected to additional band filtering, resulting in the division of frequencies into sub-frequency bands, namely theta, alpha (lower/upper alpha), beta, and gamma. After applying the band-pass filtering, the pipeline splits into two separate paths for the following steps. The primary objective of the first path (Fig. 3—solid lines and dash (dot) lines) is to establish a threshold for determining whether a section of data in non-baseline activity is contaminated and should be eliminated. Subsequently, the threshold gets employed in the second path (as depicted in Fig. 3 using solid lines) to carry out data cleaning.

In order to establish the threshold using the first path, the algorithm identifies artifacts as amplitude outliers, given that the corresponding signal is generally at least two orders of magnitude greater than the brain activity-related signal power^50^. The individual threshold can be determined by the median power of the preliminary processed baseline signal (i.e., eyes-open/closed) and then multiplying it by a safety margin factor. That says, a larger margin factor raises the threshold and increases the risk of leaving artifacts in the data, whereas a smaller margin factor lowers the threshold and more cognitively relevant EEG segments could be judged as contaminated and discarded from analysis. The value of the safety margin factor for signal power is set at 16 after exploratory checks on our dataset, which corresponds to four times the amplitude of the EEG signal. This value ensures a successful elimination of artifacts without being excessive^40^. This estimate aligns with the results of clinical research, which indicates that the average EEG potential of a person is often below $100 \upmu {\rm V}$^50^.

Artifacts are naturally unavoidable and consequently, they also contaminated the baseline data, for instance, through eye movements, which affected the average of the baseline data that was utilized as the threshold. Therefore, it was necessary to first clean the baseline data. Using the same pipeline, the artifacts and data outliers that occurred in the baseline data were detected through a moving window, with a length of 0.25 seconds and a shift of 25% of the window length. The window with data that had a median power above 16 times the median of the entire baseline session was removed. Next, the median power of the cleaned baseline was multiplied by 16 to be used as the overall threshold level for all other tasks in the protocol for the corresponding subject through the same moving window. We quantified signal quality as the proportion of data retained after artifact rejection for each participant, task, and frequency band. Supplementary Fig. S1 online shows retention rate in TTCT-IF versus DwMTs, and Supplementary Table S1 online reports group statistics (mean, standard deviation, minimum, maximum and Pearson’s r).

Eventually, the median power (medPOW) of each dataset was calculated to obtain the TRP according to Eq. (1).

1 $$\begin{aligned} TRP_{ij} = log(medPOW_{ij}(task)/medPOW_{ij}(baseline)) \end{aligned}$$

The $medPOW_{ij}$ was determined for each subject *j*, for every electrode *i*, using the processed data of a task/baseline activity. The obtained $TRP_{ij}$ value enabled the observation of the brain’s activation processes. Specifically, a TRP value greater than 0 suggested that the participant had higher activation during task engagement compared to the baseline. A TRP value below 0 indicated that the participant experienced lower activation during the task compared to the baseline.

### Statistical evaluation

Table 3 presents the three hypotheses to address the research questions.

**Table 3:** Three hypotheses to address the research questions.

Hypothesis	RQ	$H_0$ = null hypothesis	$H_a$ = alternate hypothesis
1	RQ1	TRP de/synchronization patterns measured in different frequency bands do not change under different eye conditions used as the baseline	TRP de/synchronization patterns measured in different frequency bands change under different eye conditions used as the baseline
2	RQ2-(a)	Using the eyes-open baseline condition, TRP values by bandwidth at the left-side and right-side channels within the same sketching task are the same	Using the eyes-open baseline condition, TRP values by bandwidth at the left-side and right-side channels within the same sketching task are different
3	RQ2-(b)	Using the eyes-open baseline condition, TRP values by bandwidth at each channel in TTCT-IF sketching and DwMT sketching are the same	Using the eyes-open baseline condition, TRP values by bandwidth at each channel in TTCT-IF sketching and DwMT sketching are different

For each frequency band, we first checked normality (Shapiro-Wilk) and homogeneity of variance (Bartlett) of the data in each comparison. When assumptions were met, the paired differences were analyzed with a paired t-test. Otherwise, we used the non-parametric Wilcoxon signed-rank test. To mitigate Type I error inflation from multiple comparisons, we applied the Benjamini-Hochberg false discovery rate (BH-FDR) procedure^51^, declaring significance at the corrected p-value (i.e., $q_{\text {FDR}}$) $\le 0.05$ within each comparison family, aligned with the three hypotheses. The details of the statistics reported in the Results section include $q_{\text {FDR}}$, the Wilcoxon statistic (*Z*), effect sizes for paired t-tests as Hedges’ $g_z$ (small-sample corrected Cohen’s $d_z$)^52,53^, effect sizes for signed-rank tests as matched-pairs rank-biserial correlation $r_{rb}$^54,55^. This approach ensured that the appropriate statistical test was used, depending on the distribution and variance of the data.

### Participants

A cohort of 37 participants with an engineering background had been recruited for the study, and informed consent was obtained from all of them. Four EEG datasets were deemed unsuitable for analysis due to inadequate recording caused by technological issues. The sample size for the final analysis included 33 participants. Among them, there was 1 English speaker (Spanish male, age = 28), 7 Chinese native speakers (6 female, age M=29.33, SD=5.01, 1 male, age=30), and 25 Italian speakers (6 females, age M=23.17, SD=4.92, 19 males, age M=24.95, SD= 4.94).

The participants were given a task description that was written in a language matching their cognitive abilities. The task description was translated by at least two individuals who are native speakers of each respective language. Among the 33 participants, 30 identified themselves as right-hand dominant, while the remaining 3 participants indicated no clear hand dominance despite exclusively using their right hand for writing/sketching. All participants reported no neurological or psychiatric disorders. Some samples of the sketches are presented in Supplementary Fig. S2 online.

We conducted a sample-size-based sensitivity analysis (minimum detectable effect, MDE^56,57^) for the comparisons at two-sided $\alpha = 0.05$. With 33 paired observations, the 80% power returns these thresholds: $d_z \approx 0.503$ (Hedges’ $g_z \approx 0.491$) for paired t-tests, and $r=Z/\sqrt{n_{eff}}\approx 0.488$ for Wilcoxon (for $n_{eff}=33$). This indicates that our study is sufficiently powered to detect medium or larger effects^58^. In addition, we provide the achieved (post-hoc) power based on the observed effect sizes in Supplementary Tables S2-S4 online.

The Research Ethics Committee of the Politecnico di Milano approved the described experimental protocol. All the methods were carried out in accordance with relevant guidelines and regulations.

## Results

This section is organized into two primary subsections. The first presents the results of baseline comparisons in TTCT-IF under eyes-open and eyes-closed conditions. The second focuses on comparisons of brain behavior during sketching activities in TTCT-IF and DwMT, including analyses of bilateral TRP asymmetry and channel-channel comparisons.

### EEG baseline comparisons in TTCT-IF: eyes-open versus eyes-closed

**Fig. 4:**
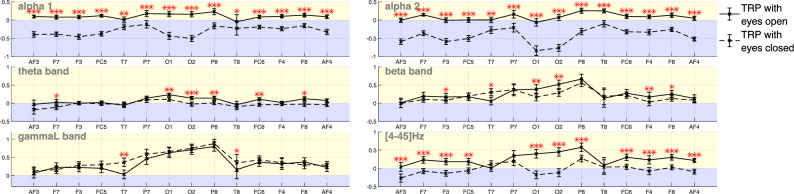
(Mean TRP patterns (group average) with error bars (standard error) across the band of alpha1, alpha2, theta, beta, lower gamma and the whole frequency band in TTCT-IF sketching, using eyes-open (solid line) and eyes-closed (dashed line) as baselines. TRP values above zero (yellow area) indicate TRP synchronization, while values below 0 (blue area) indicate TRP desynchronization. Asterisks indicate the level of statistical significance in the comparison between the two baseline conditions: $$* q_{\text {FDR}} \le 0.05, ** q_{\text {FDR}} \le 0.01, ***q_{\text {FDR}} \le 0.001$$.

Following the literature’s focus on the lower and upper alpha bands rather than the entire alpha band, this study reports the alpha band behavior in its two sub-frequency bands (alpha1 [7–10]Hz, alpha2 [10–13]Hz). Besides, the analysis also covers the other frequency bands suitable for the collected data to allow a broader comparison in the following elaborations. Figure 4 shows TRP values across different EEG channels in sub-alpha bands, theta band ([4–7] Hz), beta band ([13–30] Hz), and lower gamma band ([30–45]Hz), and the bandwidth that covers all the examined frequency bands in this study (i.e., the whole frequency band [4–45]Hz) during TTCT-IF sketching. Positive values, represented in the yellow area, indicate TRP synchronization, which reflects an increase in brain activity from baseline to the TTCT-IF sketching. Negative values, found in the blue area, indicate TRP desynchronization, suggesting a decrease in brain activity during TTCT-IF sketching compared with the baseline. The asterisk symbol denotes the rejection of the null hypothesis, which marks the statistically significant differences between TRP values obtained in the case of eyes-open baseline and eyes-closed baseline conditions. A single asterisk (*) indicates $q_{\text {FDR}}$ between 0.01 and 0.05. Two asterisks (**) denote $q_{\text {FDR}}$ between 0.001 and 0.01. Three asterisks (***) represent $q_{\text {FDR}}$ less than 0.001. There are no markers for $q_{\text {FDR}}$ greater than 0.05. Likewise, this applies to the rest of the paper. Details of the comparison statistics are presented in Table 4.

**Table 4:** Baseline condition paired comparison statistics by band and channel (eyes-open - eyes-closed).

		Channels													
		AF3	F7	F3	FC5	T7	P7	O1	O2	P8	T8	FC6	F4	F8	AF4
$q_{\text {FDR}}$	alpha1	4.26$\cdot 10^{-5}$	8.26$\cdot 10^{-5}$	2.28 $\cdot 10^{-7}$	3.62$\cdot 10^{-8}$	0.009	1.36 $\cdot 10^{-4}$	4.32$\cdot 10^{-5}$	4.50 $\cdot 10^{-5}$	1.38 $\cdot 10^{-4}$	0.013	1.21 $\cdot 10^{-4}$	1.32 $\cdot 10^{-6}$	4.41 $\cdot 10^{-4}$	2.07 $\cdot 10^{-5}$
	alpha2	2.31$\cdot 10^{-5}$	4.84 $\cdot 10^{-5}$	2.28$\cdot 10^{-7}$	4.18$\cdot 10^{-5}$	0.009	1.89 $\cdot 10^{-4}$	4.18 $\cdot 10^{-5}$	4.18 $\cdot 10^{-5}$	4.26 $\cdot 10^{-5}$	3.79 $\cdot 10^{-4}$	4.50 $\cdot 10^{-5}$	4.26 $\cdot 10^{-5}$	6.24 $\cdot 10^{-5}$	2.07 $\cdot 10^{-5}$
	theta	0.060	0.035	0.566	0.399	0.700	0.369	0.009	7.63 $\cdot 10^{-4}$	0.002	0.266	0.003	0.213	0.029	0.113
	beta	0.511	0.131	0.032	0.953	0.014	0.877	0.003	0.002	0.081	0.716	0.244	0.009	0.032	0.312
	gammaL	0.716	0.409	0.376	0.369	0.002	0.105	0.716	0.566	0.184	0.023	0.257	0.566	0.396	0.511
	[4–45]Hz	6.33 $\cdot 10^{-4}$	9.79 $\cdot 10^{-4}$	1.30 $\cdot 10^{-4}$	0.002	0.231	0.057	4.84 $\cdot 10^{-5}$	4.98 $\cdot 10^{-5}$	6.61 $\cdot 10^{-4}$	0.425	2.57 $\cdot 10^{-4}$	1.09 $\cdot 10^{-4}$	6.61 $\cdot 10^{-4}$	7.44 $\cdot 10^{-5}$
$g_z$	alpha1	–	–	1.347	1.586	–	–	–	–	–	–	–	1.316	0.740	1.024
	alpha2	–	–	1.395	–	–	–	–	–	–	–	–	–	–	–
	theta	–	–	–	0.174	–	–	–	–	–	–	–	0.269	0.433	0.309
	beta	–	–	–	–	–	–	–	–	0.357	–	–	–	–	–
	gammaL	–	–	–	–	-0.705	–	–	–	–	–	–	–	–	–
	[4–45]Hz	–	–	–	–	–	–	–	–	0.753	–	–	–	–	–
$r_{rb}$	alpha1	0.963	0.888	–	–	0.585	0.830	0.923	0.966	0.854	0.552	0.880	–	–	–
	alpha2	0.996	0.956	–	0.940	0.596	0.811	0.972	0.995	0.944	0.811	0.902	0.913	0.891	0.992
	theta	0.412	0.480	-0.133	–	-0.092	0.217	0.591	0.766	0.720	0.269	0.652	–	–	–
	beta	0.157	0.365	0.473	0.012	-0.576	0.037	0.665	0.667	–	-0.088	0.274	0.587	0.488	0.243
	gammaL	-0.084	0.202	-0.213	-0.214	–	-0.384	-0.080	-0.127	-0.310	-0.556	-0.266	-0.136	0.194	-0.153
	[4–45]Hz	0.768	0.738	0.847	0.682	-0.302	0.460	0.907	0.918	–	0.191	0.806	0.859	0.750	0.926
*Z*	alpha1	4.530	4.247	–	–	2.841	4.095	4.488	4.463	4.083	2.685	4.141	–	–	–
	alpha2	4.762	4.418	–	4.566	2.849	4.002	4.573	4.600	4.515	3.816	4.450	4.506	4.331	4.821
	theta	2.064	2.293	-0.647	–	-0.442	1.039	2.828	3.600	3.384	1.265	3.216	–	–	–
	beta	0.751	1.685	2.337	0.059	-2.664	0.168	3.178	3.291	–	-0.394	1.333	2.808	2.335	1.162
	gammaL	-0.387	0.934	-1.018	-1.039	–	-1.806	-0.402	-0.634	-1.509	-2.476	-1.293	-0.638	0.974	-0.745
	[4–45]Hz	3.671	3.527	4.115	3.260	-1.369	2.090	4.409	4.391	–	0.897	3.919	4.174	3.645	4.281

In both sub-alpha bands, participants showed TRP desynchronization across all channels in TTCT-IF sketching under the eyes-closed baseline condition. Conversely, with the eyes-open baseline, the sub-alpha bands showed an opposite variation trend (i.e., synchronization) across most channels, except for T8 in the alpha1 band and O1 in the alpha2 band. In the theta band, channels that exhibited synchronization under the eyes-closed baseline (F3, P7, O1, P8) also showed synchronization under the eyes-open condition. However, most channels that showed desynchronization under the eyes-closed baseline reversed to synchronization when using the eyes-open baseline, except for channels AF3, T7, and T8. In the beta and lower gamma bands, the synchronization pattern remained consistent across all channels under both baseline conditions. As a result of combining all the frequency bands that had different TRP patterns, the whole band exhibited a mix of desynchronization (in the frontal and occipital channels) and synchronization (in the temporal and parietal channels) under the eyes-closed baseline condition. Then, except for channel AF3 and temporal channels, the rest of the channels showed clearly a synchronization pattern under the eyes-open baseline condition.

The two baseline conditions showed statistically significant differences across all the examined bandwidths and in different channels with medium to large effect size ($|g_z|$ ranged from 0.433 to 1.586; $|r_{rb}|$ ranged from 0.473 to 0.996). The sub-alpha bands, in particular, demonstrated significant differences across all channels. The theta and beta bands showed around half of the available channels with statistically significant differences between the two baseline conditions. However, the lower gamma band exhibited statistically significant difference only at channels T7 and T8. Overall, the whole frequency band showed significant differences across all channels, except for T7 , T8 and P7.

### Sketching in TTCT-IF vs sketching in DwMT

#### Bilateral TRP asymmetry comparison

**Fig. 5:**
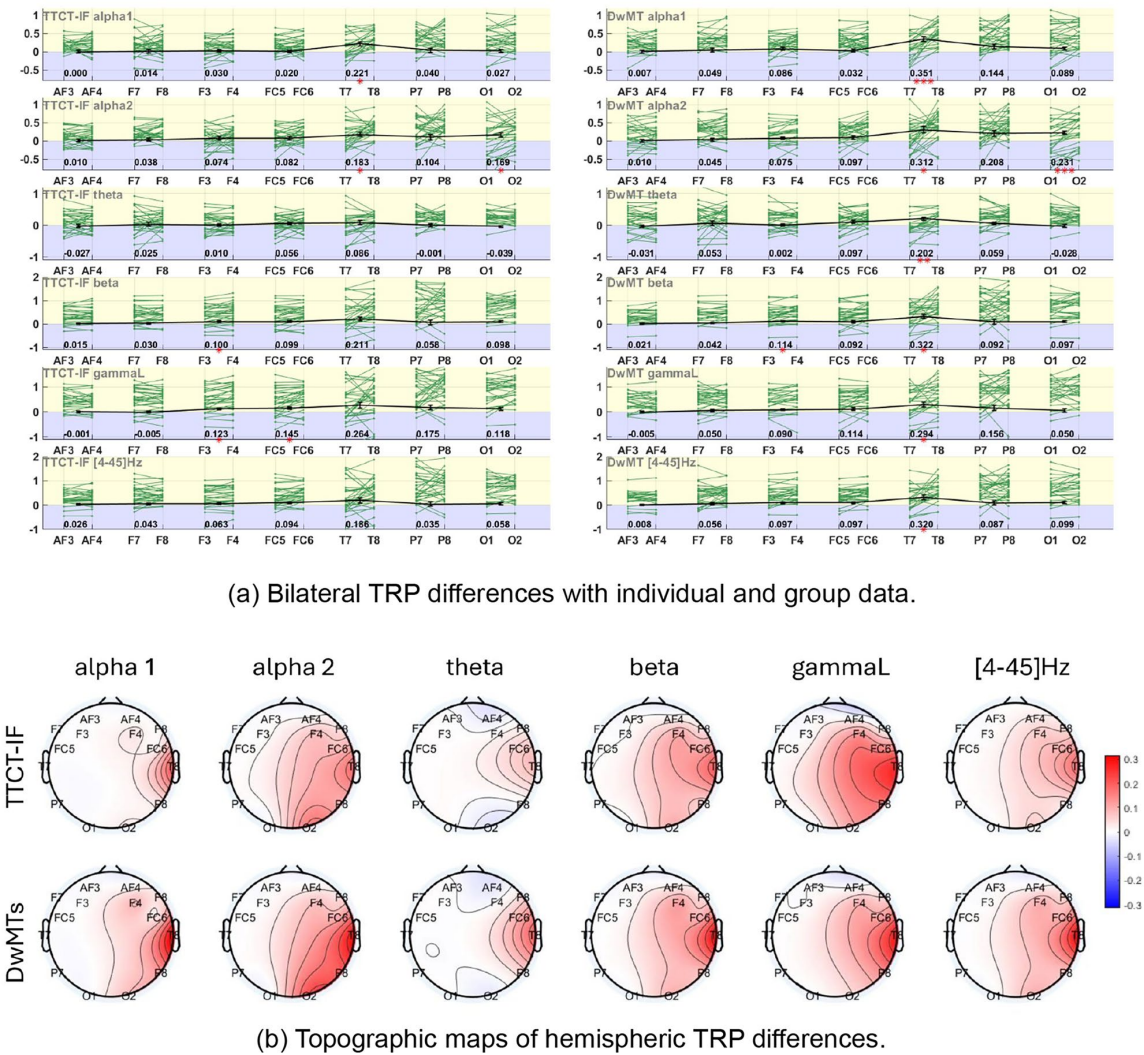
TRP bilateral differences ($TRP_{right} - TRP_{left}$) in TTCT-IF and DwMT sketching tasks across sub-alpha, theta, beta, lower gamma, and the full-band ranges. (**a**) Green lines connect individual participants’ TRP values for each bilateral channel pair; black lines indicate group-averaged differences with standard errors, with red asterisks marking significant bilateral effects. Numerical values of the bilateral differences are displayed below each channel pair. (**b**) Topographic maps of within-subject hemispheric TRP differences (left channels set to zero as a result of $TRP_{left} - TRP_{left}$; right channels show $TRP_{right} - TRP_{left}$), averaged across participants for each task and frequency band, expressed on a logarithmic scale calculated by Eq. (1). Red areas indicate higher TRP in the right hemisphere than in the left, whereas blue areas indicate higher TRP in the left hemisphere.

Figure 5a indicates the extent of differences by bandwidth between the right-side channels and their symmetrical left-side pair. Positive values, represented in the yellow area, indicate right-side channel dominance, while negative values, represented in the blue area, reflect left-side prevalence. Figure 5b presents the within-subject differences by hemisphere and averaged across participants for two tasks. The left-side channels are used as references for the right-side channels. Color indicates the extent to which the right-side channels are activated compared to the left. Table 5 shows the statistical details. Statistically significant differences between the paired channels, as presented in each task, rejected the second null hypothesis. This says that the left-side and the right-side channels (where asterisks are present in Fig. 5a) are different in each of the two sketching activities. However, the pairs with significant differences differ between the two tasks and depend on the frequency band.

**Table 5:** TRP bilateral differences ($TRP_{right} - TRP_{left}$) comparison statistics by band and channel.

		TTCT-IF							DwMTs						
		AF3 vs AF4	F7 vs F8	F3 vs F4	FC5 vs FC6	T7 vs T8	P7 vs P8	O1 vs O2	AF3 vs AF4	F7 vs F8	F3 vs F4	FC5 vs FC6	T7 vs T8	P7 vs P8	O1 vs O2
$q_{\text {FDR}}$	alpha1	0.995	0.857	0.552	0.701	0.017	0.648	0.662	0.923	0.474	0.107	0.615	6.06$\cdot 10^{-4}$	0.107	0.152
	alpha2	0.837	0.526	0.299	0.206	0.028	0.336	0.037	0.883	0.477	0.109	0.095	0.020	0.063	6.28$\cdot 10^{-4}$
	theta	0.662	0.808	0.857	0.337	0.107	0.995	0.268	0.552	0.627	0.995	0.109	0.010	0.379	0.648
	beta	0.712	0.552	0.025	0.053	0.062	0.695	0.058	0.627	0.365	0.025	0.107	0.020	0.542	0.107
	gammaL	0.995	0.942	0.019	0.032	0.086	0.210	0.080	0.951	0.477	0.076	0.107	0.028	0.214	0.627
	[4–45]Hz	0.477	0.487	0.080	0.056	0.186	0.808	0.418	0.713	0.518	0.053	0.063	0.020	0.542	0.107
$g_z$	alpha1	-0.001	0.051	0.160	0.099	0.724	0.124	0.114	0.033	0.218	0.385	0.137	1.044	–	0.347
	alpha2	0.061	0.176	0.259	0.303	0.578	0.253	0.515	0.043	0.194	0.402	0.401	0.606	0.460	0.944
	theta	-0.112	0.071	0.051	0.239	–	-0.003	-0.286	-0.153	0.129	0.009	0.366	0.828	0.237	-0.123
	beta	0.099	0.160	0.587	0.480	0.474	0.103	0.491	0.142	0.263	0.606	0.380	0.637	0.162	0.398
	gammaL	-0.003	-0.026	0.643	0.530	0.404	0.300	0.435	-0.023	0.196	–	0.396	0.591	–	0.135
	[4–45]Hz	0.202	0.190	0.446	0.472	0.315	0.068	0.220	–	0.177	0.513	0.464	0.654	0.166	0.404
$r_{rb}$	alpha1	–	–	–	–	–	–	–	–	–	–	–	–	0.428	–
	alpha2	–	–	–	–	–	–	–	–	–	–	–	–	–	–
	theta	–	–	–	–	0.455	–	–	–	–	–	–	–	–	–
	beta	–	–	–	–	–	–	–	–	–	–	–	–	–	–
	gammaL	–	–	–	–	–	–	–	–	–	0.513	–	–	0.342	–
	[4–45]Hz	–	–	–	–	–	–	–	0.117	–	–	–	–	–	–
*Z*	alpha1	–	–	–	–	–	–	–	–	–	–	–	–	2.047	–
	alpha2	–	–	–	–	–	–	–	–	–	–	–	–	–	–
	theta	–	–	–	–	2.066	–	–	–	–	–	–	–	–	–
	beta	–	–	–	–	–	–	–	–	–	–	–	–	–	–
	gammaL	–	–	–	–	–	–	–	–	–	2.330	–	–	1.635	–
	[4–45]Hz	–	–	–	–	–	–	–	0.521	–	–	–	–	–	–

Both sketching activities exhibit greater right-side dominant asymmetry in the sub-alpha bands, with higher asymmetries observed in the DwMT sketching. In the alpha1 band, for the TTCT-IF task, only the comparison between channels T7/T8 showed a statistically significant difference ($q_{\text {FDR}}=0.017$, $g_z=0.724$). The same was also found for DwMTs at T7/T8 ($q_{\text {FDR}}=6.06\cdot 10^{-4}$, $g_z=1.044$). In the alpha2 band, sketching in the TTCT-IF task showed significant differences in T7/T8 ($q_{\text {FDR}}=0.028$, $g_z=0.578$) and O1/O2 ($q_{\text {FDR}}=0.037$, $g_z=0.515$), and the DwMT sketching also exhibited statistically significant differences in these pairs, at T7/T8 ($q_{\text {FDR}}=0.020$, $g_z=0.606$), and O1/O2 ($q_{\text {FDR}}=6.28\cdot 10^{-4}$, $g_z=0.944$). For both tasks and both sub-alpha bands, the temporal area exhibited the highest bilateral TRP asymmetry among all the examined areas. Then, the caudal areas (P7/P8 in both sub-alpha bands, O1/O2 in alpha2) exhibited greater bilateral TRP asymmetries than the frontal areas (F7/F8, F3/F4, FC5/FC6). And the anterior frontal area (AF3/AF7) showed the minimum.

The results of other frequency bands provided further evidence to reject the second null hypothesis. In the theta band, the T7/T8 pair showed significant differences in DwMTs ($q_{\text {FDR}}=0.010$, $g_z=0.828$), whereas no difference was observed in TTCT-IF. Both tasks exhibited significant bilateral differences in the beta band at F3/F4, with TTCT-IF ($q_{\text {FDR}}=0.025$, $g_z=0.587$) and DwMT ($q_{\text {FDR}}=0.025$, $g_z=0.606$). Additionally, DwMTs showed a significant effect at T7/T8 ($q_{\text {FDR}}=0.020$, $g_z=0.637$). In the lower gamma band, TTCT-IF showed significant asymmetry at F3/F4 and FC5/FC6 ($q_{\text {FDR}}=0.019$, $g_z=0.643$; $q_{\text {FDR}}=0.032$, $g_z=0.530$), while DwMT showed a significant effect at T7/T8 ($q_{\text {FDR}}=0.028$, $g_z=0.591$). Over the [4–45] Hz band, DwMT also showed a significant effect at T7/T8 ($q_{\text {FDR}}=0.020$, $g_z=0.654$). Furthermore, no statistically significant differences were found at AF3/AF4, F7/F8, or P7/P8 in any frequency bands.

Overall, right-hemisphere TRP dominance was observed across various frequency ranges for both sketching activities, especially higher at the T7/T8 pair than other bilateral differences. However, the AF3/AF4 and O1/O2 pairs displayed left-side dominance in the theta band for both sketching tasks. Though less evident, the left-side dominance presented also at AF3/AF4 in the lower gamma band for both tasks. Additionally, at P7/P8 in the theta band, and F7/F8 in the lower gamma band only in the TTCT-IF sketching task.

#### Channel-channel comparison

**Fig. 6:**
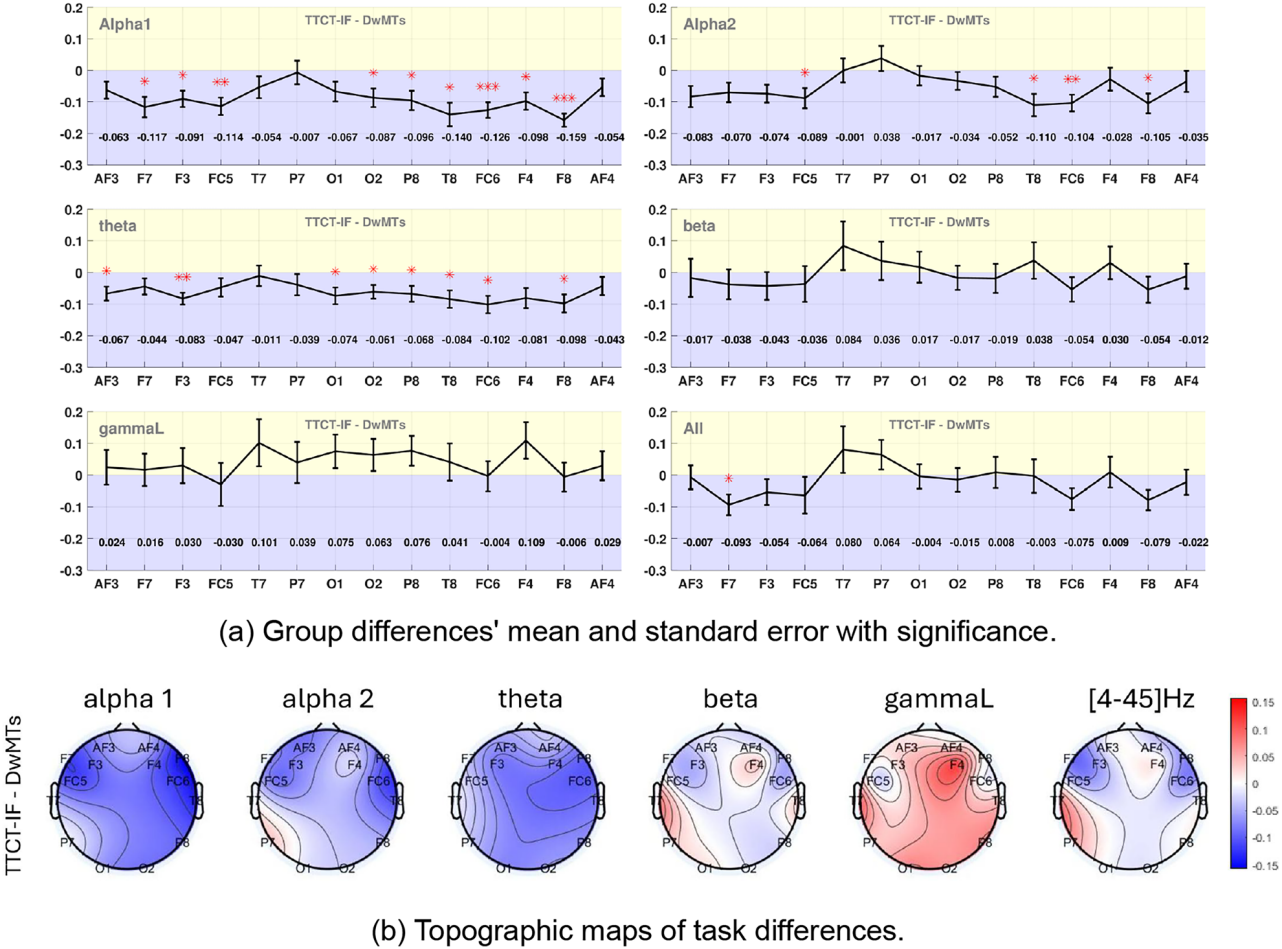
TRP differences between TTCT-IF and DwMT tasks ($TRP_{TTCT\text {-}IF} - TRP_{DwMTs}$) across frequency bands. (**a**) Group mean differences with standard errors, with red asterisks marking statistically significant task effects. (**b**) Topographic maps of within-subject task differences ($TRP_{TTCT\text {-}IF} - TRP_{DwMTs}$), averaged across participants and expressed on a logarithmic scale calculated by Eq. (1). Red areas indicate higher TRP during TTCT-IF than during DwMTs, whereas blue areas indicate higher TRP during DwMTs.

Despite the shared lateral dominance and varying location with statistically significant differences observed in the asymmetry patterns between the two sketching activities, additional distinctions between the two can be observed across various frequency bands when directly comparing them at the channel level. Figure 6a shows the statistical analysis results. Positive values (in the yellow area) indicate a higher TRP in TTCT-IF sketching, while negative values (in the blue area) represent a higher TRP during DwMTs. The mean TRP values at each channel in different frequency bands across the entire examined group are also shown in the figure. The statistically significant differences, marked with asterisks, rejected the third null hypothesis. This indicates that under the eyes-open baseline condition, TRP values at each channel for TTCT-IF sketching and DwMTs are different. The specific channels showing these differences vary across frequency bands. Moreover, Fig. 6b shows topographies of the within-subject TRP differences between the two tasks ($TRP_{TTCT\text {-}IF} - TRP_{DwMTs}$)st(TTCT-IF minus DwMTs), providing a direct visualization of these differences, and Table 6 reports the statistical details.

**Table 6:** TRP differences between TTCT-IF and DwMTs comparison statistics ($TRP_{TTCT\text {-}IF} - TRP_{DwMTs}$).

		Channels													
		AF3	F7	F3	FC5	T7	P7	O1	O2	P8	T8	FC6	F4	F8	AF4
$q_{\text {FDR}}$	alpha1	0.092	0.014	0.014	0.005	0.298	0.924	0.114	0.031	0.036	0.015	0.001	0.014	0.000	0.172
	alpha2	0.066	0.094	0.053	0.040	0.987	0.573	0.750	0.451	0.268	0.026	0.008	0.681	0.016	0.518
	theta	0.031	0.237	0.003	0.268	0.750	0.465	0.040	0.039	0.046	0.031	0.013	0.058	0.014	0.322
	beta	0.846	0.672	0.557	0.750	0.633	0.750	0.846	0.557	0.801	0.750	0.370	0.750	0.384	0.846
	gammaL	0.797	0.846	0.750	0.797	0.370	0.750	0.369	0.419	0.207	0.726	0.962	0.183	0.934	0.750
	[4–45]Hz	0.797	0.039	0.376	0.485	0.685	0.370	0.943	0.801	0.924	0.963	0.105	0.924	0.068	0.750
$g_z$	alpha1	-0.448	-0.661	-0.604	-0.725	-0.272	-0.033	-0.374	-0.518	–	–	-0.886	-0.636	-1.338	-0.343
	alpha2	-0.456	-0.402	-0.454	-0.483	-0.003	0.167	-0.094	-0.203	-0.291	-0.555	-0.687	-0.137	-0.585	-0.184
	theta	-0.565	-0.301	-0.764	-0.284	–	-0.204	-0.500	-0.508	-0.504	-0.570	-0.644	-0.460	-0.585	-0.253
	beta	–	-0.142	-0.173	-0.113	–	0.109	0.061	–	-0.073	–	-0.246	0.108	-0.241	-0.056
	gammaL	0.080	0.057	0.096	-0.078	–	0.108	0.254	0.221	–	0.124	-0.014	0.347	-0.025	0.116
	[4–45]Hz	–	-0.522	-0.234	-0.197	–	0.259	-0.021	-0.072	0.029	-0.011	-0.398	0.033	-0.442	-0.102
$r_{rb}$	alpha1	–	–	–	–	–	–	–	–	-0.561	-0.647	–	–	–	–
	alpha2	–	–	–	–	–	–	–	–	–	–	–	–	–	–
	theta	–	–	–	–	-0.116	–	–	–	–	–	–	–	–	–
	beta	-0.062	–	–	–	0.173	–	–	-0.205	–	0.110	–	–	–	–
	gammaL	–	–	–	–	0.277	–	–	–	0.359	–	–	–	–	–
	[4–45]Hz	-0.100	–	–	–	0.153	–	–	–	–	–	–	–	–	–
*Z*	alpha1	–	–	–	–	–	–	–	–	-2.684	-3.096	–	–	–	–
	alpha2	–	–	–	–	–	–	–	–	–	–	–	–	–	–
	theta	–	–	–	–	-0.529	–	–	–	–	–	–	–	–	–
	beta	-0.298	–	–	–	0.843	–	–	-0.962	–	0.542	–	–	–	–
	gammaL	–	–	–	–	1.327	–	–	–	1.744	–	–	–	–	–
	[4–45]Hz	-0.444	–	–	–	0.745	–	–	–	–	–	–	–	–	–

Absolute effect sizes at all the significant sites were medium to large ($|g_z|$ ranged from 0.483 to 1.338; in two Wilcoxon cases, $|r_{rb}|=0.561$ and 0.647.) In the alpha1 band, statistically significant differences were observed across all channels in the right hemisphere (except for AF4) and the frontal channels F7, F3 and FC5 in the left hemisphere. In the alpha2 band, fewer channels exhibited significance, including FC5, FC6, F4 and F8.

Theta band showed statistically significant differences across the entire right hemisphere except for the F4 and AF4 channel, while on the left hemisphere, the significant differences were presented only in the AF3, F3, and O1 channels. No significant between-task differences were found in the beta band or the lower gamma band at any channel. As for the whole frequency range, statistically significant differences were presented only in the left frontal area at channel F7. Notably, channels T7, P7, and AF4 did not exhibit statistically significant differences in any frequency bands.

In general, the TTCT-IF exhibited lower TRP than the DwMTs in the lower frequency bands (i.e., theta and sub-alpha bands) across all the channels, except for P7 in alpha2, while more channels showed higher TRP in the TTCT-IF in the beta and the lower gamma bands.

Given prior work showing stronger right-hemisphere activation in females for visuospatial tasks^34^, we additionally examined gender-wise differences under both baseline conditions and across sketching activities. The results showed no statistically significant gender differences in TRP across the different comparisons, except for a single effect in the between-task comparison at FC5 in the beta band ($q_{FDR}=0.037$). Detailed statistical results of the gender-wise comparisons are reported in Supplementary Tables S5 and S6 online. st(assessing paired hemispheric differences and channel-to-channel differences). The same statistical pipeline was used, except that we applied a two-sample t-test for normally distributed between-group comparisons. The results showed no statistically significant gender differences under either baseline (eyes-open: $q_{FDR_{min}}=0.106$ at F7 in alpha2, $q_{FDR_{max}}=0.989$ across most channels and bands; eyes-closed: $q_{FDR_{min}}=0.213$ at FC6 in alpha1, $q_{FDR_{max}}=0.989$ across most channels and bands), nor in hemispheric TRP asymmetries within each task (TTCT-IF: $q_{FDR_{min}}=0.301$ at multiple sites, $q_{FDR_{max}}=0.972$ at FC5/FC6 in theta; DwMTs: $q_{FDR_{min}}=0.301$ at AF3/AF4 in gammaL and F7/F8 in beta, $q_{FDR_{max}}=0.948$ at F3/F4 in alpha1). The only significant gender effect appeared in the channel-to-channel between-task difference at a single site (FC5 in beta band, $q_{FDR}=0.037$). Nevertheless, when comparing genders within each task separately, no channel-wise differences were observed (TTCT-IF: $q_{FDR_{min}}=0.106$ at F7 in alpha2, $q_{FDR_{max}}=0.978$ at O1 in alpha1 and FC5 in [4-45]Hz; DwMTs: $q_{FDR_{min}}=0.874$ at multiple sites, $q_{FDR_{max}}=0.972$ at T7 in alpha1). These additional analyses support including all participants in a single group for analysis, without distinguishing by gender.

## Discussion

The results presented in the previous section rejected all three null hypotheses. Their alternatives addressed the research questions as follows. For a cohort of engineering students, their EEG behavior, in terms of TRP de/synchronization during TTCT-IF sketching, varies depending on the baseline condition (eyes-open vs. eyes-closed) and the frequency band used for TRP estimation. Under the eyes-open baseline condition, TRP values measured during sketching in a design task and the TTCT-IF test are different. The differences can be reflected in both bilateral asymmetry and channel-to-channel comparisons depending on the frequency band analyzed.

Our results showed that desynchronization in the sub-alpha bands observed in the TTCT-IF test under the eyes-closed baseline condition aligns with findings from Jia and Zeng’s work^14^. However, when the baseline was switched to the eyes-open condition, the current result shifted from alpha desynchronization to synchronization. As Barry et al.^33^ noted, a significant alpha decrease occurs when switching from eyes-closed to eyes-open condition, particularly in the occipital region. This suggests that when we shift from a higher power (eyes-closed) baseline to a lower power (eyes-open) baseline, TRP values consequently increase. If this increase is sufficiently large, it can alter the TRP pattern from desynchronization to synchronization.

Barry’s work^33^ also noted that while the theta and beta bands exhibit a similar decreasing trend to the alpha band when transitioning from eyes-closed to eyes-open condition, the decrease in these bands is much smaller. This trend is reflected in our results, where TRP in the theta and beta bands under the eyes-closed condition is generally lower than under the eyes-open condition. The exception, where T7 showed an increase from eyes-closed to eyes-open in the beta band, was also reported in Barry’s work^33^. As a result, the TRP values in the TTCT-IF task for both theta and beta bands did not exhibit a large increase from eyes-closed to eyes-open baseline conditions as seen in the (sub-)alpha band. Channels showing desynchronization patterns in these bands under the eyes-closed condition may continue to display desynchronization when using the eyes-open baseline (e.g. AF3 in theta and beta).

Regarding lower gamma band behavior, Danko^59^ observed a gamma increase at the F3/4 channels during the transition from eyes-closed to eyes-open condition, while Geller et al.^60^ additionally reported a gamma increase in the occipital cortex. However, despite these gamma increase patterns were also presented in the current study, we observed a more pronounced gamma increase in the T7/8 channels from eyes-closed to eyes-open. The temporal area is typically associated with auditory perception^61^ and memory processing^62^, but we cannot draw firm conclusions about the cause of this observation in the current study, as we did not specifically measure these factors that might influence gamma band behavior in the temporal area. Nonetheless, our findings show that the gamma band TRP for the TTCT-IF task resulted in a synchronized pattern, and this pattern remained consistent regardless of the baseline condition used.

In addition to the dependency on the baseline eye condition, the alpha synchronization observed in the current study differs from other studies listed in Table 1. One possible explanation for this discrepancy is thatstThis difference may be attributed to the fact that those studies^15,28,30–32^, although using an eyes-open baseline, involved mental ideation rather than actual sketching. Allowing participants to physically sketch their ideas during the TTCT-IF task in the current investigation may help stthe participants reduce cognitive load compared to mental sketching. The brain has a limited capacity for holding information, and sketching on paper helps relieve this mental burden^63^. A reduction in cognitive load could lead to an increase in alpha band power^64^. Still, we emphasize that this different pattern should not be taken as definitive evidence of reduced cognitive load when switching from mental to actual sketching. Instead, it remains a post-hoc interpretation consistent with prior findings on cognitive load. Baseline state itself has a strong impact on relative alpha-band power, and in the present, investigation we cannot disentangle baseline-related effects from task-induced differences in cognitive load. Future work should consider holding the baseline constant and comparing actual and mental sketching to distinguish baseline-related from cognitive-load-related effects.

With a deeper understanding of the influence of eye conditions on TRP estimation, it becomes clear that the selection of baseline activity is crucial for interpreting results. Using an eyes-open baseline may facilitate a more direct comparison of brain activity during task performance, aligning with the natural visual environment in which sketching typically occurs. This aligns with findings in the literature that demonstrate how visual processing and attention-related neural mechanisms are more engaged in eyes-open conditions, particularly in tasks requiring visuospatial skills like sketching^15,33^. Under the eyes-open baseline condition, the current study shows that sketching activities exhibit diverse but patterned TRP bilateral asymmetries across different frequency bands. In other words, except in the lower gamma band, while the channel pairs with significant differences varied across all the investigated frequency bands, the same channel pairs that displayed significant differences in the TTCT-IF task also showed significance in the DwMTs. It is important to note that the TRP bilateral asymmetries observed in this study differ from the POW asymmetry typically reported in the literature, which is strongly related to handedness^65^. TRP asymmetry, on the other hand, reflects the degree of relative power variation differences between channels on bilateral sides from baseline (eyes-open) to the sketching activities. In this sense, side dominance refers to greater variation on one side, but not higher absolute power. Then, the pairs that share the same statistically significant differences in bilateral TRP asymmetries in both tasks can be potential indicators for sketching, while pairs showing different statistical testing results (i.e., significant in DwMTs but not in TTCT-IF at T7/T8 in the theta, beta and [4-45]Hz band; significant in TTCT-IF but not in DwMTs at F3/F4 and FC5/FC6 in the lower gamma band) may serve as indicators for differentiating between sketching in TTCT-IF and design tasks.

In design sketching, the need to visualize complex spatial relationships can lead to increased visuospatial cognitive processing, possibly higher than that required for sketching in TTCT-IT, which involves simpler graphical expressions. Design sketching engages both global and local processing as designers must conceptualize the overall structure while also attending to detailed features, such as dimensions and functional elements^16,38^. In contrast, the TTCT-IF task, which emphasizes creativity through simple geometric patterns and holistic designs, promotes a broader conceptual approach rather than detailed refinement^37^. Consistent with these task demands in visuospatial processing, our results show prominent temporal asymmetry in the sub-alpha bands and additional right occipital involvement in alpha2, with effects more evident in the DwMTs. This pattern aligns with a right-laterized temporo-parietal attention system engaged during visuospatial processing^66,67^ and with alpha2 accounts linking posterior modulation to internal attention and inhibitory control during goal-directed processing^68,69^. Theta is associated with working memory and cognitive control^70^, the significant effect at temporal sites in DwMT aligns with stronger constraint satisfaction and integrative reasoning. Additionally, gamma oscillations are often linked to the integration of visual information and perceptual binding^71,72^. Particularly, the right hemisphere tends to be more involved in global visual processing and holistic perception, while the left hemisphere is often associated with local feature processing^73^. A more evident TRP bilateral difference is therefore expected to be present in TTCT-IF sketching than in DwMTs, as shown in our results.

The direct channel-to-channel comparison between the two sketching activities provides additional insights into the distinct cognitive processes involved in design sketching versus creativity test sketching. The role of alpha synchronization in the correlation with visual creativity has been consistently highlighted in the literature^11,74^. The more creativity-related a task is, the stronger the synchronization of alpha activity^75^.

The alpha synchronization observed in both sketching activities confirmed the creativity traits necessary for both design sketching and TTCT-IF sketching. A higher level of synchronization engaged in design sketching suggests that design sketching may engage more complex creative cognitive processes, reflecting a greater involvement of creativity traits in design-oriented thinking than in the more straightforward divergent thinking promoted by TTCT-IF tasks.

However, there might be additional factors that contribute to the observed differences between the two tasks. As sketching in DwMT requires participants to generate a single design concept, while TTCT-IF prompts them to sketch multiple ideas, we expected a stronger convergent thinking process in DwMT sketching and a more divergent thinking process in TTCT-IF. Contrary to previous research, which often associates divergent thinking with greater alpha power in the right-side posterior regions^76^ as well as central area^77^, the present study observed an unexpected pattern. Specifically, the sketching activity in DwMTs (convergent sketching) displayed higher sub-alpha band power than in TTCT-IF (divergent sketching) across different brain areas, more evident in right posterior regions and also both sides frontal areas. This can be seen for different reasons. Previous research often employs tests for divergent and convergent thinking that use identical tasks, differing mainly in how unfamiliar or unusual the task is for the individual. In this study, both tasks—DwMT and TTCT-IF–were new to the participants, indicating that unfamiliarity was not the primary factor influencing brain activation. Instead, the purpose of each task likely shaped cognitive processes. In addition, it is known that sketches can stimulate the designer who might then follow up with a divergent thought on how to build on that visual stimulus^63^. This suggests that convergent sketching may not be equivalent to sketching during a convergent thinking process, but requires further verification through a more tailored experiment.

Furthermore, evidence linking bigger alpha changes to higher mental effort^78^ and to higher cognitive load in visuospatial activities^79^ suggests that increased control demands from task complexity may shape the observed alpha activity. We consider the design sketching task to be more complex, as it involves more structural and functional constraints, requires domain-specific knowledge to analyze the problem, and typically entails more execution steps than TTCT-IF. Although we simplified the design sketching task to a minimal form to match TTCT-IF in duration and modality, perceived temporal demand and task complexity may still differ across participants and have influenced brain responses. While more complex tasks tend to increase cognitive load, high cognitive load can also arise in less structurally complex tasks. Such as the requirement to generate multiple solutions in TTCT-IF may itself increase cognitive load, even if the individual sketches are simpler and the knowledge required for task execution is lower. As these factors are embedded in the task nature, the EEG differences observed likely reflect a combination of these components rather than creativity alone.

The differences in theta occipital activity between the two sketching tasks align with findings by Osipova et al.^80^, which link theta oscillations to declarative memory (i.e., a type of explicit memory for facts and events). In DwMTs, participants likely draw upon prior knowledge of mechanics and physics—designing an amphibious bike by engaging declarative memory and synthesizing learned information into a single functional concept. In contrast, TTCT-IF emphasizes divergency, with less reliance on factual knowledge and more focus on creative exploration of incomplete figures. Cavanagh and Frank^70^ further support the observed theta differences at frontal channels, correlating frontal theta activity with cognitive control, particularly in decision-making, which was more relevant in the design sketching than in the TTCT-IF task. As a supplementary check, we used the working memory control task (2-back^81^) included in our protocol. The statistics for the comparisons between 2-back and each sketching task are presented in Supplementary Fig. S3 and Table S7 online, as this is not the main focus of this work. Consistent with our interpretation, TTCT-IF did not differ significantly from 2-back in the right parietal-occipital area, whereas DwMTs did.

Engel and Fries^82^ suggested that beta oscillations are associated with maintaining sensorimotor functions. Since sketching was a constant element in both tasks, similar beta behavior were expected. This was confirmed in the direct task comparison (Fig. 6), as no channel showed a statistically significant difference in the beta band between the two tasks. Meanwhile, the DwMTs also showed a lateral TRP effect at T7/T8, consistent with reports that right temporo-parietal beta activity is engaged during attentionally demanding visuocognitive operations^82,83^.

Though bilateral TRP asymmetry in the lower gamma band showed differences between the two sketching tasks in the frontal-temporal area, this was not reflected in the channel-to-channel comparison. This could happen due to the fact that TRP asymmetry reflects relative TRP differences between hemispheres rather than the TRP power at each channel. If both sketching tasks elicit similar overall levels of gamma activity at these areas but with varying degrees of left-right balance, this would show up in asymmetry measures but not necessarily in direct channel comparisons. These findings offer a new perspective for differentiating the two sketching activities in the gamma band, highlighting how bilateral asymmetry could be a more sensitive measure for sketching in different contexts.

### Limitations

The present research only recruited individuals with an engineering background who had not previously taken part in comparable assessments of creativity and lacked experience in design using the morphological table. The possible disparities in cognitive processes between the novices and the experts were overlooked. The number of female participants was lower than the number of male subjects, which resulted in an imbalanced representation and overlooks the possible gender differences. The participants came from different nationalities and had diverse mother tongue languages. Although they conducted the experiment in their languages, and the underlying cognitive behavior should not be affected by these language differences, it might potentially influence the quality of the outcomes, which is yet to be investigated in this study. In addition to these participant-related aspects, another limitation is the absence of a control condition such as drawing-copying from the current study. Such a task could have helped isolate neural activity specific to creative/design processes from general motor and visual components of sketching. Furthermore, alpha sub-band boundaries vary in the literature. Our definition span a broad portion of the alpha range, but different sub-band cutoffs might yield different observations. The headset used in the research has a limited number of channels (14). Other devices with a larger number of channels can provide more fine-grained results.

## Conclusion

The present study showed that engineering students’ brain activity during sketching in a creativity test and a design task can be distinguished using EEG data. The results first highlighted the importance of baseline selection (eyes-open vs. eyes-closed) in design neurocognition studies. An eyes-closed baseline allows for the observation of alpha desynchronization during sketching, as reported in previous studies, while an eyes-open baseline may shift the observation toward a synchronization pattern. However, the eyes-open condition is more reflective of real-world design settings, offering a more accurate interpretation of brain activity in practical design contexts. The baseline analysis in other frequency bands from this study provided further insights into brain behavior during sketching activities.

Under the eyes-open baseline, differences between the two sketching tasks were observed through both bilateral TRP asymmetry and channel-to-channel comparisons, at brain areas varying by frequency band. In terms of bilateral TRP asymmetry, regions that showed significant differences in the creativity test sketching also exhibited differences in design sketching across most of the analyzed frequency bands, except that, in lower gamma, TTCT-IF effects were frontal (F3/F4, FC5/FC6) whereas DwMTs effects were temporal (T7/T8). The areas that exhibited significant bilateral TRP asymmetry only in design sketching were located in the temporal area (in theta and beta bands.) whereas effects present only in the creativity test occurred over the frontocentral area in the lower gamma band, suggesting these areas could act as indicators for distinguishing between design sketching and sketching in creativity tests.

In the channel-to-channel comparison, significant differences were predominantly found in the theta and sub-alpha bands, especially in the left frontal and right hemisphere regions. The beta band showed similar behavior across both tasks, indicating shared cognitive processes, as it also reflected the same bilateral TRP asymmetry. Although the lower gamma band also didn’t have any channel with significant differences between the two sketching tasks, it exhibited distinct bilateral asymmetry in the occipital area only in sketching in the creativity test in the two sketching activities, suggesting that bilateral asymmetry in the gamma band at the central-frontal to temporal area could also be a sensitive indicator further differentiating the two types of sketching activities.

The current study established a foundation for investigating the brain activity of engineers while they sketch in various design contexts and employ diverse cognitive processes. Evidence of differences in brain activation between creative tests and design activities highlights the importance of neurocognitive studies in design, which are not redundant compared to those on creativity in cognitive psychology and neuroscience. The aforementioned limitations present potential opportunities for future research. Further investigation of brain activity during other design sketching tasks is necessary to confirm the reliability of the findings. A larger sample size and varied EEG devices would be beneficial to expand the scope of the study. Comparing between non-design convergent drawing activities with design sketching tasks that mainly involve convergent thinking may shed light on the uniqueness of design thinking. Additionally, analyzing iterative micro-level transitions between divergent and convergent thinking in different sketching contexts may provide a finer-grained insight into the differences in the underlying dynamic. Through these efforts, the cognitive processes involved in sketching for various design purposes may be better explained, ultimately contributing to the formalization of a comprehensive atlas of design neurocognitive behavior, informing future sketching training and practical applications for engineering and design professionals.

## Electronic Supplementary Material

Below is the link to the electronic supplementary material.

Supplementary Material 1

## Data Availability

The datasets generated and analyzed in this study are available from the corresponding author upon reasonable request.

## References

[1] Cross, N. Natural intelligence in design. *Des. Stud.* **20**, 25–39. 10.1016/S0142-694X(98)00026-X (1999).

[2] Prats, M., Lim, S., Jowers, I., Garner, S. W. & Chase, S. Transforming shape in design: Observations from studies of sketching. *Des. Stud.* **30**, 503–520. 10.1016/j.destud.2009.04.002 (2009).

[3] Kavakli, M. & Gero, J. S. Sketching as mental imagery processing. *Des. Stud.* **22**, 347–364. 10.1016/S0142-694X(01)00002-3 (2001).

[4] Martín-Mariscal, A., Aguilar-Alejandre, M. & Peralta, M. E. Sketching and creativity: An integrated model of graphic ideation in industrial design. In *Advances in Design Engineering III* (Cavas-Martínez, F., Marín Granados, M. D. et al.. Eds.). 1067–1080, 10.1007/978-3-031-20325-1_81 (Springer, 2023).

[5] Verstijnen, I., van Leeuwen, C., Goldschmidt, G., Hamel, R. & Hennessey, J. Sketching and creative discovery. *Des. Stud.* **19**, 519–546. 10.1016/S0142-694X(98)00017-9 (1998). 10.1016/s0001-6918(98)00010-9 9708032

[6] Camba, J. D., Kimbrough, M. & Kwon, E. Conceptual product design in digital and traditional sketching environments: A comparative exploratory study. *J. Des. Res.* **16**, 131–154. 10.1504/JDR.2018.092810 (2018).

[7] Evans, M., Pei, E., Cheshire, D. & Graham, I. Digital sketching and haptic sketch modelling during product design and development. *Int. J. Prod. Dev.* **20**, 239. 10.1504/ijpd.2015.069323 (2015).

[8] Kudrowitz, B., Te, P. & Wallace, D. The influence of sketch quality on perception of product-idea creativity. *Artif. Intell. Eng. Des. Anal. Manuf.* **26**, 267–279. 10.1017/S0890060412000145 (2012).

[9] Kudrowitz, B. M. & Wallace, D. Assessing the quality of ideas from prolific, early-stage product ideation. *J. Eng. Des.* **24**, 120–139. 10.1080/09544828.2012.676633 (2013).

[10] Dietrich, A. & Kanso, R. A review of EEG, ERP, and neuroimaging studies of creativity and insight. *Psychol. Bull.* **136**, 822–48. 10.1037/a0019749 (2010). 20804237 10.1037/a0019749

[11] Pidgeon, L. M. et al. Functional neuroimaging of visual creativity: A systematic review and meta-analysis. *Brain Behav.* **6**, e00540. 10.1002/brb3.540 (2016). 27781148 10.1002/brb3.540 PMC5064346

[12] Zangeneh Soroush, M. & Zeng, Y. EEG-based study of design creativity: A review on research design, experiments, and analysis. *Front. Behav. Neurosci.*10.3389/fnbeh.2024.1331396 (2024). 39148896 10.3389/fnbeh.2024.1331396 PMC11325867

[13] Torrance, E. P. *Torrance Tests of Creative Thinking* (Personnel Press Inc, 1968).

[14] Jia, W. & Zeng, Y. EEG signals respond differently to idea generation, idea evolution and evaluation in a loosely controlled creativity experiment. *Sci. Rep.*10.1038/s41598-021-81655-0 (2021). 33483583 10.1038/s41598-021-81655-0 PMC7822831

[15] Rominger, C. et al. The creative brain in the figural domain: Distinct patterns of EEG alpha power during idea generation and idea elaboration. *Neuropsychologia* **118**, 13–19. 10.1016/j.neuropsychologia.2018.02.013 (2018). 29452125 10.1016/j.neuropsychologia.2018.02.013

[16] Rodgers, P., Green, G. & McGown, A. Using concept sketches to track design progress. *Des. Stud.* **21**, 451–464. 10.1016/S0142-694X(00)00018-1 (2000).

[17] Vieira, S., Benedek, M., Gero, J., Li, S. & Cascini, G. Design spaces and EEG frequency band power in constrained and open design. *Int. J. Des. Creativ. Innov.* **10**, 193–221. 10.1080/21650349.2022.2048697 (2022).

[18] Ferguson, E. S. *Engineering and the Mind’s Eye* (MIT Press, 1994).

[19] Brun, J., Le Masson, P. & Weil, B. Designing with sketches: The generative effects of knowledge preordering. *Des. Sci.* **2**, e13. 10.1017/dsj.2016.13 (2016).

[20] Tversky, B. What do sketches say about thinking. In *2002 AAAI Spring Symposium, Sketch Understanding Workshop, Stanford University, AAAI Technical Report SS-02-08*. Vol. 148. 151 (2002).

[21] Goel, V. *Sketches of Thought* (MIT Press, 1995).

[22] Van der Lugt, R. How sketching can affect the idea generation process in design group meetings. *Des. Stud.* **26**, 101–122. 10.1016/j.destud.2004.08.003 (2005).

[23] Schon, D. A. & Wiggins, G. Kinds of seeing and their functions in designing. *Des. Stud.* **13**, 135–156. 10.1016/0142-694X(92)90268-F (1992).

[24] Jansson, D. G. & Smith, S. M. Design fixation. *Des. Stud.* **12**, 3–11. 10.1016/0142-694X(91)90003-F (1991).

[25] Smith, S. M., Ward, T. B. & Schumacher, J. S. Constraining effects of examples in a creative generation task. *Mem. Cognit.* **21**, 837–845. 10.3758/BF03202751 (1993). 8289661 10.3758/bf03202751

[26] Razoumnikova, O. M. Functional organization of different brain areas during convergent and divergent thinking: An EEG investigation. *Cognit. Brain Res.* **10**, 11–18. 10.1016/S0926-6410(00)00017-3 (2000). 10.1016/s0926-6410(00)00017-3 10978688

[27] Jauk, E., Benedek, M. & Neubauer, A. C. Tackling creativity at its roots: Evidence for different patterns of EEG alpha activity related to convergent and divergent modes of task processing. *Int. J. Psychophysiol.* **84**, 219–225. 10.1016/j.ijpsycho.2012.02.012 (2012). 22390860 10.1016/j.ijpsycho.2012.02.012 PMC3343259

[28] Jaušovec, N. & Jaušovec, K. EEG activity during the performance of complex mental problems. *IInt. J. Psychophysiol.* **36**, 73–88. 10.1016/S0167-8760(99)00113-0 (2000). 10700625 10.1016/s0167-8760(99)00113-0

[29] van der Meer, A. L. H. & van der Weel, F. R. R. Only three fingers write, but the whole brain works: A high-density EEG study showing advantages of drawing over typing for learning. *Front. Psychol.*10.3389/fpsyg.2017.00706 (2017). 28536546 10.3389/fpsyg.2017.00706 PMC5422512

[30] Vol’f, N. V. & Tarasova, I. V. Electrophysiological parameters and the possibility of increasing imaginal creativity using monetary rewards. *Neurosci. Behav. Physiol.* **44**, 268–276. 10.1007/s11055-014-9906-5 (2014).

[31] Volf, N. V. & Tarasova, I. V. The relationships between EEG and oscillations and the level of creativity. *Hum. Physiol.* **36**, 132–138. 10.1134/S0362119710020027 (2010).

[32] Razumnikova, O. M., Volf, N. V. & Tarasova, I. V. Strategy and results: Sex differences in electrographic correlates of verbal and figural creativity. *Hum. Physiol.* **35**, 285–294. 10.1134/s0362119709030049 (2009). 19534402

[33] Barry, R. J., Clarke, A. R., Johnstone, S. J., Magee, C. A. & Rushby, J. A. EEG differences between eyes-closed and eyes-open resting conditions. *Clin. Neurophysiol.* **118**, 2765–2773. 10.1016/j.clinph.2007.07.028 (2007). 17911042 10.1016/j.clinph.2007.07.028

[34] Vieira, S., Benedek, M., Gero, J., Li, S. & Cascini, G. Brain activity in constrained and open design: The effect of gender on frequency bands. *Artif. Intell. Eng. Des. Anal. Manuf.* **36**, e6. 10.1017/S0890060421000202 (2022).

[35] Kahneman, D. *Thinking, Fast and Slow* (Farrar, Straus and Giroux, 2011).

[36] Nijstad, B. A., Dreu, C. K. W. D., Rietzschel, E. F. & Baas, M. The dual pathway to creativity model: Creative ideation as a function of flexibility and persistence. *Eur. Rev. Soc. Psychol.* **21**, 34–77. 10.1080/10463281003765323 (2010).

[37] Kim, K. H. Can we trust creativity tests? A review of the torrance tests of creative thinking (ttct). *Creativ. Res. J.* **18**, 3–14. 10.1207/s15326934crj1801_2 (2006).

[38] Ullman, D. G., Wood, S. & Craig, D. The importance of drawing in the mechanical design process. *Comput. Graph.* **14**, 263–274. 10.1016/0097-8493(90)90037-X (1990).

[39] Kannengiesser, U. & Gero, J. S. Design thinking, fast and slow: A framework for Kahneman’s dual-system theory in design. *Des. Sci.* **5**, e10. 10.1017/dsj.2019.9 (2019).

[40] Li, S., Becattini, N. & Cascini, G. Neuro-cognitive insights into engineering design: Exploring EEG predictive associations with task performance. *J. Mech. Des.* **10**(1115/1), 4066681 (2024).

[41] Peirce, J. et al. Psychopy2: Experiments in behavior made easy. *Behav. Res. Methods* **51**, 195–203. 10.3758/s13428-018-01193-y (2019). 30734206 10.3758/s13428-018-01193-y PMC6420413

[42] Heaton, R. K., Chelune, C., Talley, J., Kay, G. G. & Curtiss, G. *Wisconsin Card Sorting Test Manual: Revised and Expanded* (Psychological Assessment Resources Inc, 1993).

[43] Bobrov, P. et al. Brain-computer interface based on generation of visual images. *PLoS ONE* **6**, e20674. 10.1371/journal.pone.0020674 (2011). 21695206 10.1371/journal.pone.0020674 PMC3112189

[44] Amjad, I. et al. Therapeutic effects of aerobic exercise on EEG parameters and higher cognitive functions in mild cognitive impairment patients. *Int. J. Neurosci.* **129**, 551–562. 10.1080/00207454.2018.1551894 (2019). 30929591 10.1080/00207454.2018.1551894

[45] Lukaevi, F., Becattini, N., Periši, M. M. & Škec, S. Differences in engineers’ brain activity when cad modelling from isometric and orthographic projections. *Sci. Rep.* **13**. 10.1038/s41598-023-36823-9 (2023). 10.1038/s41598-023-36823-9 PMC10272240 37322108

[46] Krumm, G., Arán Filippetti, V., Catanzariti, M. & Mateos, D. M. Exploring the neural basis of creativity: EEG analysis of power spectrum and functional connectivity during creative tasks in school-aged children. *Front. Comput. Neurosci.* **19**. 10.3389/fncom.2025.1548620 (2025). 10.3389/fncom.2025.1548620 PMC11937046 40145081

[47] Makeig, S., Bell, A., Jung, T.-P. & Sejnowski, T. J. Independent component analysis of electroencephalographic data. *Adv. Neural Inf. Process* (1995).

[48] Berg, P. & Scherg, M. Dipole modelling of eye activity and its application to the removal of eye artefacts from the EEG and MEG. *Clin. Phys. Physiol. Meas.* **12**, 49–54. 10.1088/0143-0815/12/a/010 (1991). 1778052 10.1088/0143-0815/12/a/010

[49] Borga, M. & Knutsson, H. A canonical correlation approach to blind source separation. In *Technical Report, Report LiU-IMT-EX-0062 Department of Biomedical Engineering, Linkping University* (2001).

[50] Stern, J. M. *Atlas of EEG Patterns*. 2 Ed. (Lippincott Williams and Wilkins, 2013).

[51] Benjamini, Y. & Hochberg, Y. Controlling the false discovery rate: A practical and powerful approach to multiple testing. *J. R. Stat. Soc. Ser.B (Methodol.)* **57**, 289–300 (1995).

[52] Hedges, L. V. Distribution theory for glass’s estimator of effect size and related estimators. *J. Educ. Stat.* **6**, 107–128. 10.3102/10769986006002107 (1981).

[53] Hedges, L. V. & Olkin, I. *Statistical Methods for Meta-Analysis* (Academic Press, 1985).

[54] Cureton, E. E. Rank-biserial correlation. *Psychometrika* **21**, 287–290. 10.1007/BF02289138 (1956).

[55] Willson, V. L. Critical values of the rank-biserial correlation coefficient. *Educ. Psychol. Meas.* **36**, 297–300. 10.1177/001316447603600207 (1976).

[56] Bloom, H. S. Minimum detectable effects: A simple way to report the statistical power of experimental designs. *Eval. Rev.* **19**, 547–556. 10.1177/0193841X9501900504 (1995).

[57] Lakens, D. Sample size justification. *Collab. Psychol.* **8**. 10.1525/collabra.33267 (2022).

[58] Cohen, J. *Statistical Power Analysis for the Behavioral Sciences* (Elsevier, 1977).

[59] Danko, S. G. The reflection of different aspects of brain activation in the electroencephalogram: Quantitative electroencephalography of the states of rest with the eyes open and closed. *Hum. Physiol.* **32**, 377–388. 10.1134/s0362119706040013 (2006). 16910068

[60] Geller, A. S. et al. Eye closure causes widespread low-frequency power increase and focal gamma attenuation in the human electrocorticogram. *Clin. Neurophysiol.* **125**, 1764–1773. 10.1016/j.clinph.2014.01.021 (2014). 24631141 10.1016/j.clinph.2014.01.021 PMC4127381

[61] Edwards, E., Soltani, M., Deouell, L. Y., Berger, M. S. & Knight, R. T. High gamma activity in response to deviant auditory stimuli recorded directly from human cortex. *J. Neurophysiol.* **94**, 4269–4280. 10.1152/jn.00324.2005 (2005). 16093343 10.1152/jn.00324.2005

[62] Fell, J. et al. Rhinal-hippocampal theta coherence during declarative memory formation: Interaction with gamma synchronization?. *European Journal of Neuroscience* **17**, 1082–1088. 10.1046/j.1460-9568.2003.02522.x (2003). 12653984 10.1046/j.1460-9568.2003.02522.x

[63] Bilda, Z. & Gero, J. Does sketching off-load visuo-spatial working memory? In *Studying Designers’05*. Vol. 5. 145–159 (2005).

[64] Stipacek, A., Grabner, R., Neuper, C., Fink, A. & Neubauer, A. Sensitivity of human EEG alpha band desynchronization to different working memory components and increasing levels of memory load. *Neurosci. Lett.* **353**, 193–196. 10.1016/j.neulet.2003.09.044 (2003). 14665414 10.1016/j.neulet.2003.09.044

[65] Ocklenburg, S. et al. Beyond frontal alpha: Investigating hemispheric asymmetries over the EEG frequency spectrum as a function of sex and handedness. *Lateral. Asymm. Body Brain Cognit.* **24**, 505–524. 10.1080/1357650x.2018.1543314 (2018). 10.1080/1357650X.2018.1543314 30388061

[66] Corbetta, M. & Shulman, G. L. Control of goal-directed and stimulus-driven attention in the brain. *Nat. Rev. Neurosci.* **3**, 201–215. 10.1038/nrn755 (2002). 11994752

[67] Thut, G., Nietzel, A., Brandt, S. A. & Pascual-Leone, A. -band electroencephalographic activity over occipital cortex indexes visuospatial attention bias and predicts visual target detection. *J. Neurosci.* **26**, 9494–9502. 10.1523/JNEUROSCI.0875-06.2006 (2006). 16971533 10.1523/JNEUROSCI.0875-06.2006 PMC6674607

[68] Klimesch, W. Alpha-band oscillations, attention, and controlled access to stored information. *Trends Cognit. Sci.* **16**, 606–617. 10.1016/j.tics.2012.10.007 (2012). 23141428 10.1016/j.tics.2012.10.007 PMC3507158

[69] Jensen, O. & Mazaheri, A. Shaping functional architecture by oscillatory alpha activity: Gating by inhibition. *Front. Hum. Neurosci.* **4**. 10.3389/fnhum.2010.00186 (2010). 10.3389/fnhum.2010.00186 PMC2990626 21119777

[70] Cavanagh, J. F. & Frank, M. J. Frontal theta as a mechanism for cognitive control. *Trends Cognit. Sci.* **18**, 414–421. 10.1016/j.tics.2014.04.012 (2014). 24835663 10.1016/j.tics.2014.04.012 PMC4112145

[71] Spydell, J. D. & Sheer, D. E. Effect of problem solving on right and left hemisphere 40 hertz EEG activity. *Psychophysiology* **19**, 420–425. 10.1111/j.1469-8986.1982.tb02497.x (1982). 7122780 10.1111/j.1469-8986.1982.tb02497.x

[72] Tallon-Baudry, C. & Bertrand, O. Oscillatory gamma activity in humans and its role in object representation. *Trends Cognit. Sci.* **3**, 151–162. 10.1016/S1364-6613(99)01299-1 (1999). 10322469 10.1016/s1364-6613(99)01299-1

[73] Hellige, J. Hemispheric asymmetry for visual information processing. *Acta Neurobiol. Exp.* **56**, 485–497. 10.55782/ane-1996-1151 (1996). 8787209

[74] Fink, A. & Benedek, M. EEG alpha power and creative ideation. *Neurosci. Biobehav. Rev.* **44**, 111–123. 10.1016/j.neubiorev.2012.12.002 (2014). 23246442 10.1016/j.neubiorev.2012.12.002 PMC4020761

[75] Fink, A., Benedek, M., Grabner, R. H., Staudt, B. & Neubauer, A. C. Creativity meets neuroscience: Experimental tasks for the neuroscientific study of creative thinking. *Methods* **42**, 68–76. 10.1016/j.ymeth.2006.12.001 (2007). 17434417 10.1016/j.ymeth.2006.12.001

[76] Shemyakina, N. V., Danko, S. G., Nagornova, Z. V., Starchenko, M. G. & Bechtereva, N. P. Changes in the power and coherence spectra of the EEG rhythmic components during solution of a verbal creative task of overcoming a stereotype. *Hum. Physiol.* **33**, 524–530. 10.1134/s0362119707050027 (2007). 18038659

[77] Mölle, M., Marshall, L., Wolf, B., Fehm, H. L. & Born, J. EEG complexity and performance measures of creative thinking. *Psychophysiology* **36**, 95–104. 10.1017/S0048577299961619 (1999). 10098384 10.1017/s0048577299961619

[78] Keil, A., Mussweiler, T. & Epstude, K. Alpha-band activity reflects reduction of mental effort in a comparison task: A source space analysis. *Brain Res.* **1121**, 117–127. 10.1016/j.brainres.2006.08.118 (2006). 17010944 10.1016/j.brainres.2006.08.118

[79] Lukaevi, F., Becattini, N. & Škec, S. Identifying the electroencephalography features for measuring cognitive load in computer-aided design. *J. Mech. Des.* **147**, 121403. 10.1115/1.4068746 (2025).

[80] Osipova, D. et al. Theta and gamma oscillations predict encoding and retrieval of declarative memory. *J. Neurosci.* **26**, 7523–7531. 10.1523/JNEUROSCI.1948-06.2006 (2006). 16837600 10.1523/JNEUROSCI.1948-06.2006 PMC6674196

[81] Kirchner, W. K. Age differences in short-term retention of rapidly changing information. *J. Exp. Psychol.* **55**, 352–358. 10.1037/h0043688 (1958). 13539317 10.1037/h0043688

[82] Engel, A. K. & Fries, P. Beta-band oscillations—Signalling the status quo?. *Curr. Opin. Neurobiol.* **20**, 156–165. 10.1016/j.conb.2010.02.015 (2010). 20359884 10.1016/j.conb.2010.02.015

[83] Park, J., Kim, H., Sohn, J.-W., Choi, J.-R. & Kim, S.-P. EEG beta oscillations in the temporoparietal area related to the accuracy in estimating others’ preference. *Front. Hum. Neurosci.*10.3389/fnhum.2018.00043 (2018). 29479312 10.3389/fnhum.2018.00043 PMC5811502

