# Supplementary material for: Neurocognitive differences in sketching between design tasks and creativity tests
Source: Sci Rep. 2026 Feb 20;16:9964. doi: 10.1038/s41598-026-38735-w (PMC13021921; doi:10.1038/s41598-026-38735-w)
Supplement: Supplementary file 1 — Supplementary Material 1 [file 41598_2026_38735_MOESM1_ESM.pdf]

Neurocognitive differences in sketching between design tasks and creativity tests

Shumin Li[1,*], Gaetano Cascini[1], and Niccolò Becattini[1]

[1]Politecnico di Milano, Department of Mechanical Engineering, Milano, 20158, Italy
[*]shumin.li@polimi.it

Supplementary Information

	alpha1		alpha2		theta		beta		gammaL		[4–45] Hz	
	TTCT	DwMT	TTCT	DwMT	TTCT	DwMT	TTCT	DwMT	TTCT	DwMT	TTCT	DwMT
mean	0.566	0.715	0.577	0.715	0.727	0.932	0.642	0.827	0.525	0.683	0.727	0.932
std	0.131	0.175	0.152	0.203	0.146	0.084	0.163	0.170	0.129	0.144	0.146	0.084
max	0.840	0.959	0.863	0.990	0.967	0.997	0.924	0.994	0.801	0.914	0.967	0.997
min	0.244	0.223	0.232	0.209	0.442	0.679	0.252	0.207	0.303	0.419	0.442	0.679
Pearson's r	0.564		0.652		0.382		0.557		0.542		0.382	

Table S1. Proportion of retained EEG data after artifact rejection by task and frequency band (mean, SD, max, min across participants).

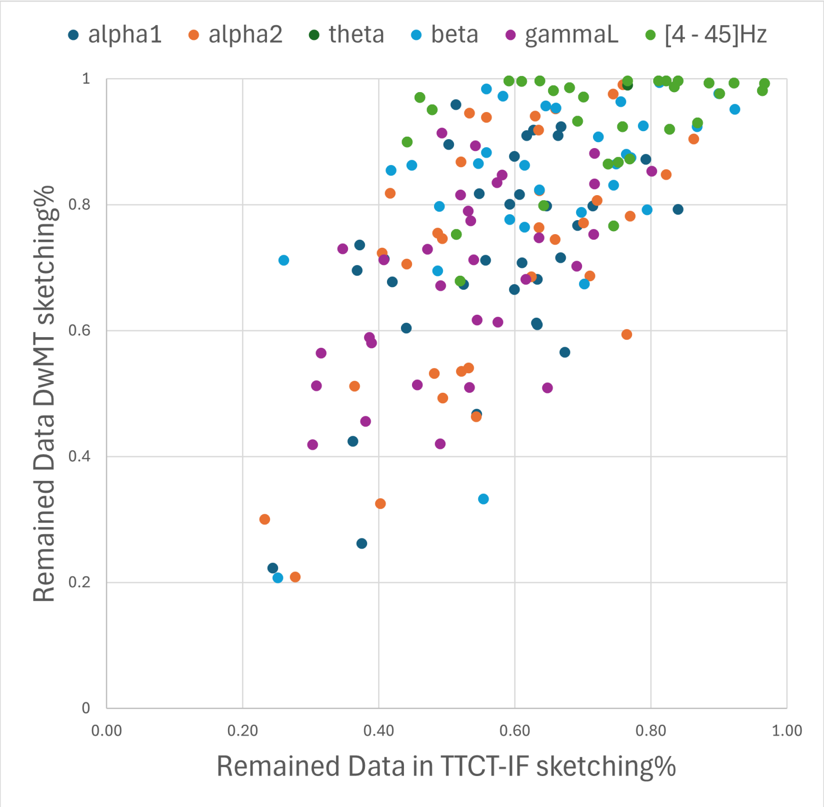

Figure S1. Proportion of retained data after artifact rejection for DwMT sketching versus TTCT-IF sketching, plotted per participant and frequency band. Points near the upper right indicate high retention in both tasks.

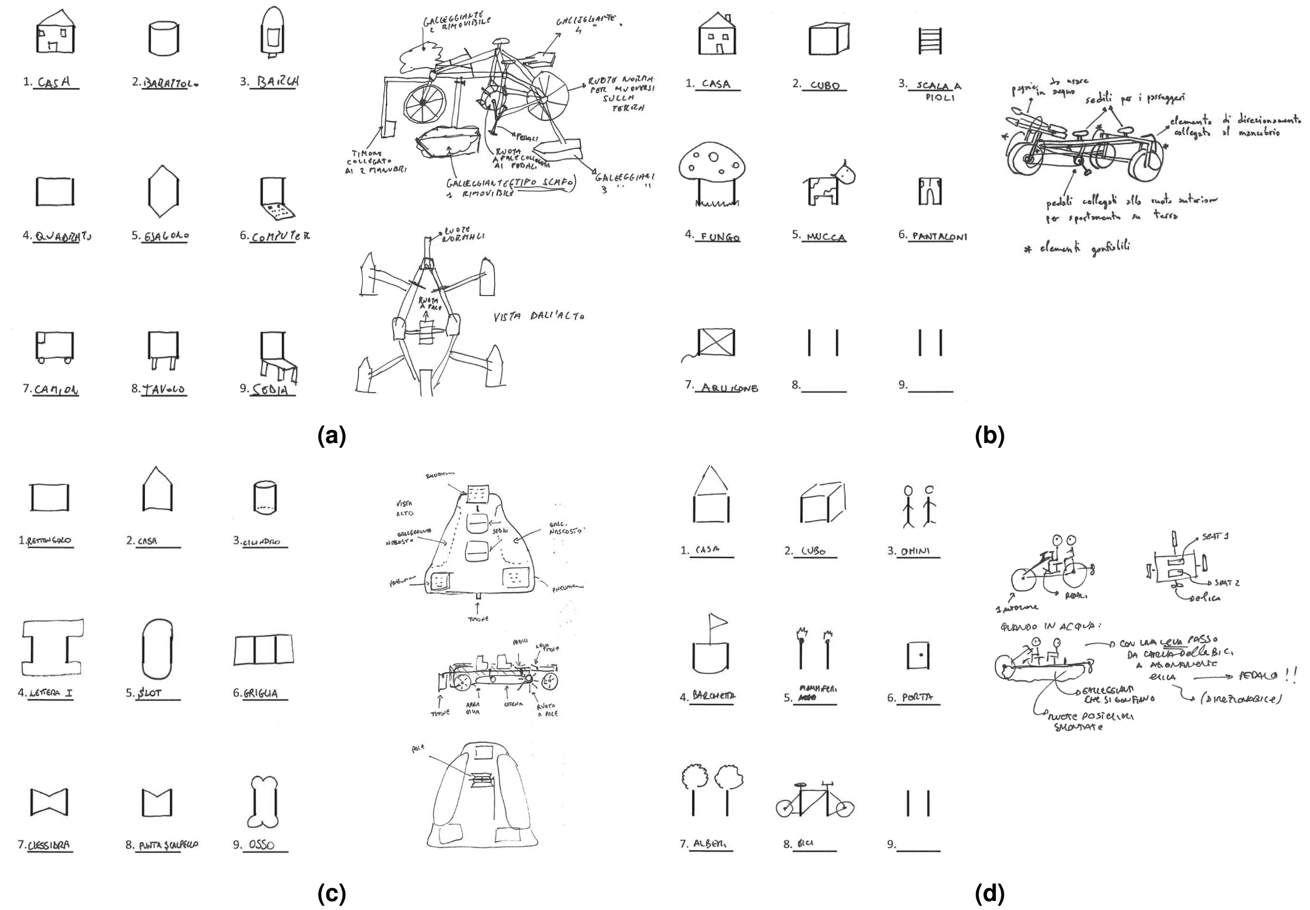

Figure S2. Sample sketches. Each pair shows sketches by the same participant, with TTCT-IF on the left and DwMT on the right.

		Channels													
		AF3	F7	F3	FC5	T7	P7	O1	O2	P8	T8	FC6	F4	F8	AF4
p_{raw}	alpha1	$5.90\cdot10^{-6}$	$2.16\cdot10^{-5}$	$8.14\cdot10^{-9}$	$4.31\cdot10^{-10}$	0.004	$4.22\cdot10^{-5}$	$7.20\cdot10^{-6}$	$8.07\cdot10^{-6}$	$4.45\cdot10^{-5}$	0.007	$3.46\cdot10^{-5}$	$6.30\cdot10^{-8}$	$1.63\cdot10^{-4}$	$1.48\cdot10^{-6}$
	alpha2	$1.92\cdot10^{-6}$	$9.98\cdot10^{-6}$	$6.78\cdot10^{-9}$	$4.97\cdot10^{-6}$	0.004	$6.29\cdot10^{-5}$	$4.80\cdot10^{-6}$	$4.23\cdot10^{-6}$	$6.34\cdot10^{-6}$	$1.35\cdot10^{-4}$	$8.57\cdot10^{-6}$	$6.59\cdot10^{-6}$	$1.49\cdot10^{-5}$	$1.43\cdot10^{-6}$
	theta	0.039	0.022	0.518	0.337	0.658	0.299	0.005	$3.18\cdot10^{-4}$	$7.14\cdot10^{-4}$	0.206	0.001	0.155	0.018	0.078
	beta	0.453	0.092	0.019	0.953	0.008	0.866	0.001	$9.98\cdot10^{-4}$	0.054	0.694	0.183	0.005	0.020	0.245
	gammaL	0.699	0.350	0.309	0.299	$8.38\cdot10^{-4}$	0.071	0.688	0.526	0.131	0.013	0.196	0.524	0.330	0.456
	[4–45]Hz	$2.41\cdot10^{-4}$	$4.20\cdot10^{-4}$	$3.87\cdot10^{-5}$	0.001	0.171	0.037	$1.04\cdot10^{-5}$	$1.13\cdot10^{-5}$	$2.66\cdot10^{-4}$	0.370	$8.88\cdot10^{-5}$	$2.99\cdot10^{-5}$	$2.67\cdot10^{-4}$	$1.86\cdot10^{-5}$
q_{FDR}	alpha1	$4.26\cdot10^{-5}$	$8.26\cdot10^{-5}$	$2.28\cdot10^{-7}$	$3.62\cdot10^{-8}$	0.009	$1.36\cdot10^{-4}$	$4.32\cdot10^{-5}$	$4.50\cdot10^{-5}$	$1.38\cdot10^{-4}$	0.013	$1.21\cdot10^{-4}$	$1.32\cdot10^{-6}$	$4.41\cdot10^{-4}$	$2.07\cdot10^{-5}$
	alpha2	$2.31\cdot10^{-5}$	$4.84\cdot10^{-5}$	$2.28\cdot10^{-7}$	$4.18\cdot10^{-5}$	0.009	$1.89\cdot10^{-4}$	$4.18\cdot10^{-5}$	$4.18\cdot10^{-5}$	$4.26\cdot10^{-5}$	$3.79\cdot10^{-4}$	$4.50\cdot10^{-5}$	$4.26\cdot10^{-5}$	$6.24\cdot10^{-5}$	$2.07\cdot10^{-5}$
	theta	0.060	0.035	0.566	0.399	0.700	0.369	0.009	$7.63\cdot10^{-4}$	0.002	0.266	0.003	0.213	0.029	0.113
	beta	0.511	0.131	0.032	0.953	0.014	0.877	0.003	0.002	0.081	0.716	0.244	0.009	0.032	0.312
	gammaL	0.716	0.409	0.376	0.369	0.002	0.105	0.716	0.566	0.184	0.023	0.257	0.566	0.396	0.511
	[4–45]Hz	$6.33\cdot10^{-4}$	$9.79\cdot10^{-4}$	$1.30\cdot10^{-4}$	0.002	0.231	0.057	$4.84\cdot10^{-5}$	$4.98\cdot10^{-5}$	$6.61\cdot10^{-4}$	0.425	$2.57\cdot10^{-4}$	$1.09\cdot10^{-4}$	$6.61\cdot10^{-4}$	$7.44\cdot10^{-5}$
g_z	alpha1	/	/	1.347	1.586	/	/	/	/	/	/	/	1.316	0.740	1.024
	alpha2	/	/	1.395	/	/	/	/	/	/	/	/	/	/	/
	theta	/	/	/	0.174	/	/	/	/	/	/	/	0.269	0.433	0.309
	beta	/	/	/	/	/	/	/	/	0.357	/	/	/	/	/
	gammaL	/	/	/	/	-0.705	/	/	/	/	/	/	/	/	/
	[4–45]Hz	/	/	/	/	/	/	/	/	0.753	/	/	/	/	/
r_{rb}	alpha1	0.963	0.888	/	/	0.585	0.830	0.923	0.966	0.854	0.552	0.880	/	/	/
	alpha2	0.996	0.956	/	0.940	0.596	0.811	0.972	0.995	0.944	0.811	0.902	0.913	0.891	0.992
	theta	0.412	0.480	-0.133	/	-0.092	0.217	0.591	0.766	0.720	0.269	0.652	/	/	/
	beta	0.157	0.365	0.473	0.012	-0.576	0.037	0.665	0.667	/	-0.088	0.274	0.587	0.488	0.243
	gammaL	-0.084	0.202	-0.213	-0.214	/	-0.384	-0.080	-0.127	-0.310	-0.556	-0.266	-0.136	0.194	-0.153
	[4–45]Hz	0.768	0.738	0.847	0.682	-0.302	0.460	0.907	0.918	/	0.191	0.806	0.859	0.750	0.926
Z	alpha1	4.530	4.247	/	/	2.841	4.095	4.488	4.463	4.083	2.685	4.141	/	/	/
	alpha2	4.762	4.418	/	4.566	2.849	4.002	4.573	4.600	4.515	3.816	4.450	4.506	4.331	4.821
	theta	2.064	2.293	-0.647	/	-0.442	1.039	2.828	3.600	3.384	1.265	3.216	/	/	/
	beta	0.751	1.685	2.337	0.059	-2.664	0.168	3.178	3.291	/	-0.394	1.333	2.808	2.335	1.162
	gammaL	-0.387	0.934	-1.018	-1.039	/	-1.806	-0.402	-0.634	-1.509	-2.476	-1.293	-0.638	0.974	-0.745
	[4–45]Hz	3.671	3.527	4.115	3.260	-1.369	2.090	4.409	4.391	/	0.897	3.919	4.174	3.645	4.281
$1-\beta$	alpha1	0.995	0.989	$\sim$1.000	$\sim$1.000	0.811	0.984	0.994	0.994	0.983	0.766	0.985	$\sim$1.000	0.986	$\sim$1.000
	alpha2	0.997	0.993	$\sim$1.000	0.995	0.813	0.979	0.996	0.996	0.995	0.968	0.994	0.995	0.991	0.998
	theta	0.541	0.631	0.099	0.157	0.073	0.180	0.807	0.950	0.923	0.244	0.895	0.292	0.681	0.423
	beta	0.117	0.392	0.647	0.050	0.759	0.053	0.888	0.908	0.493	0.068	0.266	0.802	0.646	0.213
	gammaL	0.067	0.154	0.175	0.180	0.953	0.439	0.069	0.097	0.326	0.697	0.253	0.098	0.164	0.116
	[4–45]Hz	0.957	0.942	0.984	0.903	0.278	0.552	0.993	0.992	0.980	0.146	0.975	0.987	0.954	0.990

Table S2. Baseline condition paired comparison statistics by band and channel (eyes-open - eyes-closed).

		TTCT-IF							DwMTs						
		AF3 vs AF4	F7 vs F8	F3 vs F4	FC5 vs FC6	T7 vs T8	P7 vs P8	O1 vs O2	AF3 vs AF4	F7 vs F8	F3 vs F4	FC5 vs FC6	T7 vs T8	P7 vs P8	O1 vs O2
p_{raw}	alpha1	0.995	0.775	0.383	0.576	$8.13\cdot10^{-4}$	0.498	0.526	0.858	0.271	0.042	0.439	$7.21\cdot10^{-6}$	0.041	0.065
	alpha2	0.737	0.338	0.149	0.093	0.004	0.172	0.006	0.810	0.285	0.045	0.029	0.002	0.015	$1.50\cdot10^{-5}$
	theta	0.528	0.694	0.774	0.177	0.039	0.989	0.131	0.388	0.459	0.961	0.045	$3.45\cdot10^{-4}$	0.207	0.501
	beta	0.594	0.382	0.003	0.010	0.014	0.562	0.013	0.469	0.195	0.003	0.038	0.001	0.361	0.039
	gammaL	0.987	0.886	0.001	0.005	0.026	0.097	0.023	0.905	0.288	0.020	0.037	0.004	0.102	0.470
	[4–45]Hz	0.290	0.302	0.022	0.011	0.082	0.702	0.234	0.603	0.327	0.010	0.016	0.002	0.359	0.037
	alpha1	0.995	0.857	0.552	0.701	0.017	0.648	0.662	0.923	0.474	0.107	0.615	$6.06\cdot10^{-4}$	0.107	0.152
	alpha2	0.837	0.526	0.299	0.206	0.028	0.336	0.037	0.883	0.477	0.109	0.095	0.020	0.063	$6.28\cdot10^{-4}$
q_{FDR}	alpha1	0.995	0.857	0.552	0.701	0.017	0.648	0.662	0.923	0.474	0.107	0.615	$6.06\cdot10^{-4}$	0.107	0.152
	alpha2	0.837	0.526	0.299	0.206	0.028	0.336	0.037	0.883	0.477	0.109	0.095	0.020	0.063	$6.28\cdot10^{-4}$
	theta	0.662	0.808	0.857	0.337	0.107	0.995	0.268	0.552	0.627	0.995	0.109	0.010	0.379	0.648
	beta	0.712	0.552	0.025	0.053	0.062	0.695	0.058	0.627	0.365	0.025	0.107	0.020	0.542	0.107
	gammaL	0.995	0.942	0.019	0.032	0.086	0.210	0.080	0.951	0.477	0.076	0.107	0.028	0.214	0.627
	[4–45]Hz	0.477	0.487	0.080	0.056	0.186	0.808	0.418	0.713	0.518	0.053	0.063	0.020	0.542	0.107
g_z	alpha1	-0.001	0.051	0.160	0.099	0.724	0.124	0.114	0.033	0.218	0.385	0.137	1.044	/	0.347
	alpha2	0.061	0.176	0.259	0.303	0.578	0.253	0.515	0.043	0.194	0.402	0.401	0.606	0.460	0.944
	theta	-0.112	0.071	0.051	0.239	/	-0.003	-0.286	-0.153	0.129	0.009	0.366	0.828	0.237	-0.123
	beta	0.099	0.160	0.587	0.480	0.474	0.103	0.491	0.142	0.263	0.606	0.380	0.637	0.162	0.398
	gammaL	-0.003	-0.026	0.643	0.530	0.404	0.300	0.435	-0.023	0.196	/	0.396	0.591	/	0.135
	[4–45]Hz	0.202	0.190	0.446	0.472	0.315	0.068	0.220	/	0.177	0.513	0.464	0.654	0.166	0.404
r_{rb}	alpha1	/	/	/	/	/	/	/	/	/	/	/	/	0.428	/
	alpha2	/	/	/	/	/	/	/	/	/	/	/	/	/	/
	theta	/	/	/	/	0.455	/	/	/	/	/	/	/	/	/
	beta	/	/	/	/	/	/	/	/	/	/	/	/	/	/
	gammaL	/	/	/	/	/	/	/	/	/	0.513	/	/	0.342	/
	[4–45]Hz	/	/	/	/	/	/	/	0.117	/	/	/	/	/	/
Z	alpha1	/	/	/	/	/	/	/	/	/	/	/	/	2.047	/
	alpha2	/	/	/	/	/	/	/	/	/	/	/	/	/	/
	theta	/	/	/	/	2.066	/	/	/	/	/	/	/	/	/
	beta	/	/	/	/	/	/	/	/	/	/	/	/	/	/
	gammaL	/	/	/	/	/	/	/	/	/	2.330	/	/	1.635	/
	[4–45]Hz	/	/	/	/	/	/	/	0.521	/	/	/	/	/	/
$1-\beta$	alpha1	0.050	0.059	0.137	0.085	0.955	0.102	0.095	0.054	0.191	0.539	0.118	$\sim$1.000	0.535	0.458
	alpha2	0.062	0.156	0.299	0.389	0.858	0.273	0.813	0.056	0.184	0.529	0.601	0.909	0.705	0.999
	theta	0.095	0.067	0.059	0.268	0.542	0.050	0.324	0.136	0.112	0.050	0.524	0.980	0.238	0.101
	beta	0.082	0.138	0.880	0.756	0.716	0.088	0.732	0.109	0.248	0.889	0.555	0.925	0.146	0.551
	gammaL	0.050	0.052	0.937	0.834	0.622	0.381	0.643	0.052	0.182	0.644	0.561	0.860	0.373	0.109
	[4–45]Hz	0.180	0.174	0.649	0.742	0.414	0.066	0.217	0.082	0.161	0.768	0.699	0.921	0.147	0.564

Table S3. TRP bilateral differences (right-left) comparison statistics by band and channel.

		Channels													
		AF3	F7	F3	FC5	T7	P7	O1	O2	P8	T8	FC6	F4	F8	AF4
p_{raw}	alpha1	0.030	0.001	0.001	$2.59{\cdot}10^{-4}$	0.131	0.854	0.041	0.006	0.007	0.002	$1.98{\cdot}10^{-5}$	0.001	$2.70{\cdot}10^{-8}$	0.064
	alpha2	0.020	0.031	0.014	0.010	0.987	0.355	0.588	0.247	0.113	0.004	$4.67{\cdot}10^{-4}$	0.446	0.002	0.302
	theta	0.006	0.096	$1.08{\cdot}10^{-4}$	0.115	0.597	0.260	0.010	0.009	0.012	0.005	$9.22{\cdot}10^{-4}$	0.017	0.002	0.146
	beta	0.766	0.432	0.338	0.524	0.399	0.553	0.736	0.336	0.683	0.588	0.177	0.562	0.201	0.763
	gammaL	0.663	0.753	0.598	0.664	0.185	0.548	0.171	0.224	0.081	0.493	0.939	0.070	0.889	0.531
	[4–45]Hz	0.657	0.008	0.192	0.277	0.456	0.185	0.909	0.687	0.869	0.952	0.036	0.858	0.021	0.571
q_{FDR}	alpha1	0.092	0.014	0.014	0.005	0.298	0.924	0.114	0.031	0.036	0.015	$8.30{\cdot}10^{-4}$	0.014	$2.27{\cdot}10^{-6}$	0.172
	alpha2	0.066	0.094	0.053	0.040	0.987	0.573	0.750	0.451	0.268	0.026	0.008	0.681	0.016	0.518
	theta	0.031	0.237	0.003	0.268	0.750	0.465	0.040	0.039	0.046	0.031	0.013	0.058	0.014	0.322
	beta	0.846	0.672	0.557	0.750	0.633	0.750	0.846	0.557	0.801	0.750	0.370	0.750	0.384	0.846
	gammaL	0.797	0.846	0.750	0.797	0.370	0.750	0.369	0.419	0.207	0.726	0.962	0.183	0.934	0.750
	[4–45]Hz	0.797	0.039	0.376	0.485	0.685	0.370	0.943	0.801	0.924	0.963	0.105	0.924	0.068	0.750
g_z	alpha1	-0.448	-0.661	-0.604	-0.725	-0.272	-0.033	-0.374	-0.518	/	/	-0.886	-0.636	-1.338	-0.343
	alpha2	-0.456	-0.402	-0.454	-0.483	-0.003	0.167	-0.094	-0.203	-0.291	-0.555	-0.687	-0.137	-0.585	-0.184
	theta	-0.565	-0.301	-0.764	-0.284	/	-0.204	-0.500	-0.508	-0.504	-0.570	-0.644	-0.460	-0.585	-0.253
	beta	/	-0.142	-0.173	-0.113	/	0.109	0.061	/	-0.073	/	-0.246	0.108	-0.241	-0.056
	gammaL	0.080	0.057	0.096	-0.078	/	0.108	0.254	0.221	/	0.124	-0.014	0.347	-0.025	0.116
	[4–45]Hz	/	-0.522	-0.234	-0.197	/	0.259	-0.021	-0.072	0.029	-0.011	-0.398	0.033	-0.442	-0.102
r_{rb}	alpha1	/	/	/	/	/	/	/	/	-0.561	-0.647	/	/	/	/
	alpha2	/	/	/	/	/	/	/	/	/	/	/	/	/	/
	theta	/	/	/	/	-0.116	/	/	/	/	/	/	/	/	/
	beta	-0.062	/	/	/	0.173	/	/	-0.205	/	0.110	/	/	/	/
	gammaL	/	/	/	/	0.277	/	/	/	0.359	/	/	/	/	/
	[4–45]Hz	-0.100	/	/	/	0.153	/	/	/	/	/	/	/	/	/
	alpha1	/	/	/	/	/	/	/	/	-2.684	-3.096	/	/	/	/
	alpha2	/	/	/	/	/	/	/	/	/	/	/	/	/	/
	theta	/	/	/	/	-0.529	/	/	/	/	/	/	/	/	/
	beta	-0.298	/	/	/	0.843	/	/	-0.962	/	0.542	/	/	/	/
	gammaL	/	/	/	/	1.327	/	/	/	1.744	/	/	/	/	/
	[4–45]Hz	-0.444	/	/	/	0.745	/	/	/	/	/	/	/	/	/
$1-\beta$	alpha1	0.603	0.934	0.924	0.980	0.324	0.054	0.543	0.816	0.766	0.872	0.998	0.925	1.000	0.462
	alpha2	0.668	0.590	0.710	0.761	0.050	0.149	0.083	0.208	0.353	0.855	0.967	0.116	0.898	0.174
	theta	0.830	0.384	0.990	0.349	0.083	0.199	0.761	0.775	0.739	0.835	0.945	0.691	0.915	0.304
	beta	0.060	0.120	0.156	0.096	0.134	0.089	0.063	0.161	0.068	0.084	0.268	0.088	0.244	0.060
	gammaL	0.071	0.061	0.081	0.071	0.264	0.090	0.273	0.225	0.415	0.103	0.051	0.445	0.052	0.094
	[4–45]Hz	0.073	0.782	0.253	0.188	0.116	0.259	0.051	0.068	0.053	0.050	0.566	0.053	0.656	0.086

Table S4. TRP differences between TTCT-IF and DwMTs comparison statistics. (TTCT-IF minus DwMTs)

The results of gender-wise comparisons under both baseline conditions and across sketching activities are shown in Table S5 and S6. The same statistical pipeline as described in the main content was also used for this examination, except that we applied a two-sample t-test for normally distributed between-group comparisons. The results showed no statistically significant gender differences under either baseline (eyes-open: $q_{FDR_{min}} = 0.106$ at F7 in alpha2, $q_{FDR_{max}} = 0.989$ across most channels and bands; eyes-closed: $q_{FDR_{min}} = 0.213$ at FC6 in alpha1, $q_{FDR_{max}} = 0.989$ across most channels and bands), nor in hemispheric TRP asymmetries within each task (TTCT-IF: $q_{FDR_{min}} = 0.301$ at multiple sites, $q_{FDR_{max}} = 0.972$ at FC5/FC6 in theta; DwMTs: $q_{FDR_{min}} = 0.301$ at AF3/AF4 in gammaL and F7/F8 in beta, $q_{FDR_{max}} = 0.948$ at F3/F4 in alpha1). The only significant gender effect appeared in the channel-to-channel between-task difference at a single site (FC5 in beta band, $q_{FDR} = 0.037$). Nevertheless, when comparing genders within each task separately, no channel-wise differences were observed (TTCT-IF: $q_{FDR_{min}} = 0.106$ at F7 in alpha2, $q_{FDR_{max}} = 0.978$ at O1 in alpha1 and FC5 in [4-45]Hz; DwMTs: $q_{FDR_{min}} = 0.874$ at multiple sites, $q_{FDR_{max}} = 0.972$ at T7 in alpha1). These additional analyses support including all participants in a single group for analysis, without distinguishing by gender.

Task / contrast	Band	AF3	F7	F3	FC5	T7	P7	O1	O2	P8	T8	FC6	F4	F8	AF4
TTCT-IF (eyes-open)	alpha1	0.989	0.989	0.989	0.989	0.989	0.989	0.989	0.989	0.725	0.989	0.989	0.989	0.989	0.989
	alpha2	0.989	0.106	0.989	0.989	0.989	0.989	0.989	0.989	0.989	0.989	0.989	0.989	0.725	0.989
	theta	0.989	0.989	0.989	0.989	0.989	0.989	0.989	0.989	0.989	0.989	0.989	0.725	0.989	0.989
	beta	0.989	0.989	0.989	0.989	0.989	0.989	0.989	0.989	0.989	0.989	0.989	0.989	0.989	0.989
	gammaL	0.989	0.989	0.989	0.989	0.989	0.989	0.989	0.989	0.989	0.989	0.989	0.989	0.989	0.989
	4–45	0.989	0.989	0.989	0.989	0.989	0.989	0.989	0.989	0.989	0.989	0.989	0.989	0.989	0.989
TTCT-IF (eyes-closed)	alpha1	0.989	0.669	0.989	0.989	0.635	0.989	0.989	0.989	0.922	0.989	0.213	0.989	0.989	0.989
	alpha2	0.989	0.989	0.989	0.989	0.989	0.989	0.989	0.989	0.989	0.984	0.989	0.669	0.989	0.989
	theta	0.989	0.989	0.989	0.989	0.989	0.989	0.989	0.989	0.989	0.989	0.989	0.989	0.989	0.989
	beta	0.989	0.989	0.989	0.989	0.989	0.989	0.989	0.989	0.989	0.989	0.989	0.989	0.991	0.989
	gammaL	0.989	0.989	0.989	0.989	0.989	0.989	0.989	0.989	0.989	0.989	0.989	0.989	0.989	0.989
	4–45	0.989	0.989	0.989	0.989	0.989	0.989	0.989	0.989	0.989	0.989	0.989	0.989	0.989	0.989
TTCT-IF − DwMTs	alpha1	0.903	0.186	0.389	0.911	0.226	0.142	0.911	0.408	0.944	0.229	0.615	0.376	0.615	0.672
	alpha2	0.408	0.408	0.680	0.935	0.680	0.142	0.615	0.903	0.922	0.623	0.497	0.408	0.680	0.459
	theta	0.944	0.459	0.697	0.944	0.944	0.944	0.835	0.389	0.490	0.944	0.274	0.186	0.615	0.408
	beta	0.911	0.186	0.142	**0.037**	0.359	0.835	0.680	0.186	0.697	0.298	0.229	0.944	0.376	0.835
	gammaL	0.777	0.331	0.086	0.142	0.389	0.825	0.944	0.935	0.935	0.805	0.173	0.995	0.997	0.376
	4–45	0.985	0.454	0.086	0.142	0.376	0.490	0.274	0.229	0.821	0.142	0.142	0.889	0.186	0.459

Table S5. FDR-corrected q values for gender-wise comparisons (female vs. male) of TRP at each channel under the eyes-open and eyes-closed baseline for TTCT-IF, and of task differences (TTCT-IF − DwMTs) across frequency bands. The only q value below 0.05 is highlighted in bold.

Task	Band	AF4–AF3	F8–F7	F4–F3	FC6–FC5	T8–T7	P8–P7	O2–O1
TTCT-IF	alpha1	0.815	0.858	0.815	0.858	0.876	0.772	0.876
	alpha2	0.764	0.858	0.301	0.770	0.764	0.815	0.876
	theta	0.797	0.876	0.301	0.972	0.770	0.764	0.555
	beta	0.301	0.764	0.764	0.858	0.861	0.815	0.770
	gammaL	0.301	0.858	0.301	0.861	0.903	0.770	0.764
	4–45	0.858	0.884	0.772	0.861	0.772	0.815	0.301
DwMTs	alpha1	0.861	0.605	0.948	0.909	0.858	0.770	0.797
	alpha2	0.815	0.861	0.845	0.770	0.555	0.884	0.861
	theta	0.903	0.905	0.555	0.763	0.858	0.845	0.876
	beta	0.764	0.301	0.651	0.597	0.858	0.858	0.858
	gammaL	0.301	0.845	0.343	0.597	0.815	0.797	0.797
	4–45	0.764	0.861	0.764	0.770	0.861	0.815	0.876

Table S6. FDR-corrected q values for gender-wise comparisons (female vs. male) of hemispheric TRP asymmetries (right − left) under the eyes-open baseline for TTCT-IF and DwMTs across frequency bands.

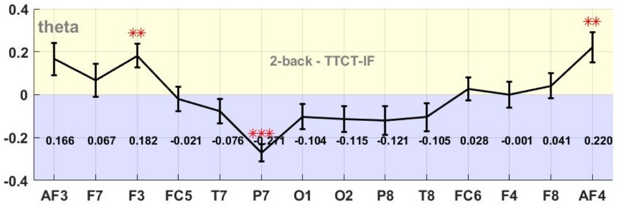

(a) TRP differences between 2-back and TTCT-IF: 2-back minus TTCT-IF.

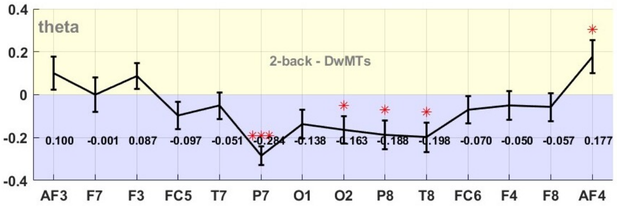

(b) TRP differences between 2-back and DwMTs: 2-back minus DwMTs.

Figure S3. TRP differences between 2-back against TTCT-IF and DwMTs.

	2-back - TTCT-IF (Theta band)													
Channels	AF3	F7	F3	FC5	T7	P7	O1	O2	P8	T8	FC6	F4	F8	AF4
p_{raw}	0.038	0.389	0.003	0.717	0.194	$3.14 \cdot 10^{-7}$	0.088	0.067	0.082	0.120	0.611	0.991	0.491	0.004
q_{FDR}	0.133	0.545	0.018	0.773	0.302	$4.39 \cdot 10^{-6}$	0.176	0.176	0.176	0.210	0.713	0.991	0.625	0.018
g_z	0.380	0.155	0.560	-0.065	-0.226	-1.273	-0.319	-0.339	-0.338	-0.300	0.087	-0.002	0.118	0.531
$1-\beta$	0.556	0.135	0.882	0.064	0.251	1.000	0.400	0.454	0.414	0.340	0.079	0.050	0.104	0.858
	2-back - DwMTs (Theta band)													
Channels	AF3	F7	F3	FC5	T7	P7	O1	O2	P8	T8	FC6	F4	F8	AF4
p_{raw}	0.207	0.990	0.158	0.141	0.410	$7.55 \cdot 10^{-7}$	0.049	0.016	0.009	0.008	0.275	0.448	0.387	0.030
q_{FDR}	0.256	0.998	0.201	0.183	0.472	$1.27 \cdot 10^{-5}$	0.070	0.025	0.016	0.014	0.335	0.501	0.457	0.044
g_z	0.222	-0.002	0.250	-0.265	-0.154	-1.174	-0.359	-0.455	-0.503	-0.527	-0.194	-0.139	-0.149	0.386
$1-\beta$	0.239	0.050	0.289	0.310	0.127	1.000	0.511	0.696	0.767	0.790	0.189	0.115	0.136	0.595

Table S7. TRP differences between 2-back vs DwMTs and 2-back vs TTCT-IF comparison statistics. (2-back minus DwMTs; 2-back minus TTCT-IF)
